# Plant-based dietary patterns, genetic predisposition and risk of colorectal cancer: a prospective study from the UK Biobank

**DOI:** 10.1186/s12967-023-04522-8

**Published:** 2023-09-27

**Authors:** Fubin Liu, Yanling Lv, Yu Peng, Yating Qiao, Peng Wang, Changyu Si, Xixuan Wang, Jianxiao Gong, Huijun Zhou, Ming Zhang, Liangkai Chen, Fangfang Song

**Affiliations:** 1 Department of Epidemiology and Biostatistics, Key Laboratory of Molecular Cancer Epidemiology, Tianjin’s Clinical Research Center for Cancer, National Clinical Research Center for Cancer, Tianjin Medical University Cancer Institute and Hospital, Tianjin, 300060 China; 2 Department of Nutrition and Food Hygiene, Hubei Key Laboratory of Food Nutrition and Safety, Ministry of Education Key Lab of Environment and Health, School of Public Health, Tongji Medical College, Huazhong University of Science and Technology, Wuhan, 430030 China; 3 Comprehensive Management Department of Occupational Health, Shenzhen Prevention and Treatment Center for Occupational Diseases, Shenzhen, 518020 China

**Keywords:** Plant-based diet indices, Diet quality, Colorectal cancer, Polygenic risk score, UK Biobank

## Abstract

**Background:**

Plant-based dietary patterns may affect colorectal cancer (CRC) related outcomes, while risks differ in the quality of plant foods. We aimed to examine the association of plant-based diet quality with risks of CRC incidence and mortality and whether this association was modified by genetic risk.

**Methods:**

This prospective cohort study included 186,675 participants free of cancer when the last dietary recall was completed. We calculated three plant-based diet indices (PDIs), i.e., the overall plant-based diet index (PDI), the healthful plant-based diet index (hPDI), and the unhealthful plant-based diet index (uPDI) representing adherence to plant-based diets with diverse quality. Genetic risk was characterized using a weighted polygenic risk score (PRS), capturing overall risk variants associated with CRC. Hazard ratios (HR) and 95% confidential intervals (CI) were estimated by the cause-specific Cox proportional hazards model.

**Results:**

Over a follow-up of 9.5 years, 2163 cases and 466 deaths from CRC were documented. The HR of CRC incidence was 0.88 (95% CI, 0.81–0.96) and 0.91 (95% CI, 0.84–0.99) per 10-score increase in PDI and hPDI, respectively. Compared to the lowest quartile, PDI, hPDI, and uPDI in the highest quartile were associated with a 13% decrease, a 15% decrease, and a 14% increase in risk of incident CRC, respectively. We found a joint association of genetic risk and PDIs with incident CRC, with the highest hazard observed in those carrying higher PRS and adhering to lower-quality PDIs. The inverse association of PDI and hPDI with CRC mortality was pronounced in males.

**Conclusions:**

Our results suggested that better adherence to overall and healthful plant-based diets was associated with a lower risk of CRC, whereas an unhealthful plant-based diet was associated with a higher CRC risk. Consumption of a higher-quality plant-based diet combined with decreased genetic risk conferred less susceptibility to CRC. Our findings highlighted the importance of food quality when adhering to a plant-based dietary pattern for CRC prevention in the general population.

**Supplementary Information:**

The online version contains supplementary material available at 10.1186/s12967-023-04522-8.

## Background

More than 1.9 million new colorectal cancer (CRC) cases and 935,000 deaths from CRC were estimated to occur in 2020 worldwide, ranking third and second in incidence and mortality, respectively [1]. Despite the considerable reduction in incidence and mortality ascribed to screening and improved treatment, CRC is often diagnosed at advanced clinical stages. Therefore, identifying and reducing modifiable risk factors are attractive primary prevention strategies to counter the escalating global rise of CRC.

The potential health effects of plant-based diets have been increasingly recognized and ascribed to their environmental sustainability benefits [2, 3]. However, not all plant-based foods were beneficial to CRC. High intakes of whole grains, fruits, vegetables, and fiber were associated with a low risk of CRC [4–7], whereas less nutrient-dense plant foods, including refined grains, fruit juices, and sugar-sweetened beverages, contributed to an increased CRC risk [8–11]. To better represent the quality of plant foods, studies recently have developed three plant-based diet indices (PDIs), i.e., an overall plant-based diet index (PDI), a healthful plant-based diet index (hPDI), and an unhealthful plant-based index (uPDI), to examine their association with various chronic diseases and mortality [12, 13]. However, given the limited study regions and inconsistent findings on CRC [14–19], evidence from large population-based studies with a prospective design is warranted.

The concept of “gene × lifestyle interaction” has presumed that modifiable lifestyle factors may yield different effects on complex diseases depending on inherited genetic susceptibility [20]. Several CRC-associated loci have been identified in genome-wide association studies [21]. However, no studies have examined the interaction between plant-based diet patterns and genetic predisposition on CRC prevention. Therefore, the present study aimed to prospectively investigate the association of PDIs with the risk of CRC in a larger general population from the UK Biobank and to explore whether such association would be modified by the genetic predisposition of CRC.

## Methods

### Study design and setting

The UK Biobank recruited more than 0.5 million participants aged 37–73 years from the general population between 2006 and 2010, and detailed information on study design, implementation, and data acquisition can be found at https://www.ukbiobank.ac.uk [22]. Participants attended one of 22 assessment centers across England, Scotland, and Wales. They completed a touch-screen questionnaire, a face-to-face interview with a nurse, and a series of physical measurements, and provided biological samples. The date and cause of hospital admissions were obtained through record linkages to health episode statistics (England and Wales) and Scottish morbidity records (Scotland). The UK Biobank study was approved by the North West Multi-centre Research Ethics Committee (REC reference for the UK Biobank 11/NW/0382), and all participants provided written informed consent.

### Study population

We included participants with at least one dietary assessment and available genetic data, and excluded those with implausible total energy intake (TEI, < 800 or > 5000 kcal/day in males and < 500 or > 4000 kcal/day in females) and diagnosed cancers (except for non-melanoma skin cancer) when dietary information collection was completed. Finally, 186,675 participants were included in the PDIs analysis; 174,261 were included in the analysis for PDIs and genetic risk after excluding those not of European descent, with incomplete genetic data, mismatch between self-reported and genetic sex, outliers for heterozygosity or missing rate, sex chromosome aneuploidy, and close kinship (Additional file 1: Fig. S1).

### Outcomes

The primary outcome was incident CRC, and the secondary outcome was CRC mortality. The detailed definition for diagnosis of overall CRC and CRC by anatomical subsites (proximal colon cancer, distal colon cancer, and rectal cancer) was described according to hospital inpatient records, cancer registry data, and death registry data linked to the UK Biobank based on the International Classification of Diseases, Ninth Revision (ICD-9) and Tenth Revision (ICD-10) codes, as well as self-reported data fields with the choice-, disease- or procedure-specific codes (Additional file 1: Table S1). Proximal colon cancers included those found in the cecum, appendix, ascending colon, hepatic flexure, transverse colon, and splenic flexure (C18.0-18.5); distal colon cancers in the descending (C18.6) and sigmoid (C18.7) colon; and rectal cancer in the rectosigmoid junction (C19) and rectum (C20). The time-to-event was calculated from the last dietary assessment to the date of CRC diagnosis, death, loss to follow-up, or censorship (30 September 2021 for England, 31 July 2021 for Scotland, and 28 February 2018 for Wales), whichever came first.

### Dietary assessment

Dietary information in the UK Biobank was collected using the Oxford WebQ, which has been validated with an interviewer-administered 24-h recall [23] and biomarkers [24], based on a 24-h dietary recall questionnaire. The consumption of more than 200 common foods and more than 30 types of beverages during the previous 24 h was collected. Participants who completed at least one 24-h dietary assessment were included. For those who completed twice or more, the intake of each food item was calculated as the means of intake answered across all dietary assessments.

We calculated the PDI, hPDI, and uPDI using established methods (containing 18 food groups) to assess the adherence to overall, healthful, and unhealthful plant-based diets, respectively [25, 26], except vegetable oils which were not available in the UK Biobank dataset. Thus, we classified food items into 17 groups and further into larger categories of healthy plant foods, less healthy plant foods and animal foods. Intake of each food group was ranked into quintiles and given positive (Q1 to Q5 received 1 to 5) or reverse (Q1 to Q5 received 5 to 1) scores (detailed in Additional file 1: Table S2). The final scores of 3 food categories and 17 food groups constructing three PDIs were presented in Additional file 1: Table S3.

### Polygenic risk score for CRC

The detail of genotyping, imputation, and quality control of genetic data in the UK Biobank has been discussed elsewhere [27]. We calculated the global polygenic risk score (PRS) for CRC based on an up-to-date genome-wide association study reporting 95 single-nucleotide polymorphisms (SNPs) significantly associated with CRC in participants of European descent [21]. The effect size of each SNP (β-coefficient) and other related information were shown in Additional file 1: Table S4. The PRS for CRC was calculated by summing the risk allele numbers of each SNP weighted by the effect size to CRC: PRS = (β_1_ × SNP_1_ + β_2_ × SNP_2_ + …+β_n_ × SNP_n_) * (N/sum of β-coefficient), where SNP_n_ was the risk allele number of each SNP.

### Covariates

Sociodemographic factors (age at the last dietary assessment, sex, ethnicity, and educational qualifications) and lifestyle factors (alcohol intake frequency, smoking status, and physical activity) were self-reported at the baseline assessment. Townsend deprivation index was applied to indicate socioeconomic status, with higher scores equating to higher socioeconomic deprivation [28]. Alcohol intake frequency was classified as daily or almost daily, three or four times a week, once or twice a week, one to three times a month, special occasions only, and never. Smoking status was categorized as current smoker, former smoker, and non-smoker. Three levels of physical activity were proposed to classify populations (low, moderate, and high) based on the International Physical Activity Questionnaire guidelines [29]. Body mass index (BMI) was calculated as weight (kg) divided by the square of height (m) and classified as < 18.5, 18.5 to 24.9, 25.0 to 29.9, and ≥ 30.0 kg/m^2^. TEI was calculated based on their answers to the dietary questionnaire [30].

### Statistical analyses

The PDI, hPDI, and uPDI scores were sorted in ascending order and classified by quartiles (Q1-Q4) using three breakpoints, i.e., P25, P50, and P75. We estimated the associations of three categorical PDIs with CRC incidence and mortality using a cause-specific Cox proportional hazards regression model with time-to-event as the timescale. The results were presented as hazard ratios (HRs) and 95% confidence intervals (CIs). The proportional hazards assumption was tested by the Schoenfeld residual method and satisfied. Missing values of covariates were treated as dummy variables. We successively adjusted for age and sex, ethnicity, education, Townsend deprivation index, BMI, alcohol frequency, smoking status, physical activity, TEI, PRS for CRC, first 10 principal components of ancestry, and genotype measurement batch. The PDIs were also treated as continuous variables, and HRs per 10-score increment were reported. To investigate the dose-response association between PDIs and CRC risk, we performed restricted cubic splines (RCS) fitted by Cox proportional hazards regression to flexibly model the CRC risk distributed by PDIs. We further investigated the association between PDIs and the incidence of CRC at different anatomical subsites.

We estimated the associations of PRS with CRC risk using a cause-specific Cox proportional hazards regression model. Then we conducted stratified analysis by CRC-PRS tertiles to assess the associations between PDIs tertiles and CRC risk among individuals with different genetic risks. Multiplicative interactions were tested by including a PDIs × PRS term in the fully adjusted model. We also estimated the joint association of PDIs and genetic risk with CRC by defining a combined variable according to the tertiles of genetic risk and PDIs (9 categories).

We conducted subgroup analyses stratified by sex in the incidence and mortality analysis, and further by age, Townsend deprivation index, BMI, alcohol frequency, smoking status, and physical activity in the incidence analysis. Multiplicative interactions were tested by including a “PDIs × covariates” term in the fully adjusted model.

For secondary analyses, we (1) conducted sensitivity analyses by excluding individuals with less than 2 years of follow-up to minimize the reverse casualty and using sub-distribution hazard models for competing risk; (2) examined the overall and sex-stratified association of three food categories (healthy plant foods, less healthy plant foods, and animal foods) with the CRC risk by adding the values in each food category together to understand which food category played a key role; (3) examined the PDIs-CRC associations after modifying the PDI and hPDI by assigning a positive score to the beneficial animal foods (dairy products and seafood) ascertained by the inverse association with CRC reported by the previous literatures [31, 32].

All analyses were performed using SAS version 9.4 (SAS Institute, USA) and R software (The R Foundation, http://www.r-project.org, version 4.0.2). A level of < 0.05 for two-sided *P* values was considered statistically significant.

## Results

### Characteristics of study population

The main baseline characteristics of participants by PDI, hPDI, and uPDI groups are shown in Table 1, Additional file 1: Tables S5 and S6, respectively. Among 186,675 cancer-free participants at baseline, the PDI ranged from 24 to 77, the hPDI ranged from 29 to 82, and the uPDI ranged from 28 to 79. Participants with higher PDI and hPDI but lower uPDI tended to be older, female, well-educated, non-current smokers, physically active, and with lower alcohol intake, TEI, and BMI.

**Table 1:** Baseline characteristics of 186,675 participants by PDI groups

	Overall plant-based diet index (PDI)			
	Q1	Q2	Q3	Q4
Range, scores	24–46	47–49	50–53	54–77
Number of participants	44,202	51,430	39,446	51,597
Age, mean (SD), years	57.5 (8.1)	58.2 (8.0)	58.5 (7.9)	58.4 (8.0)
Male, n (%)	23,760 (53.8)	23,952 (46.6)	16,964 (43.0)	20,746 (40.2)
Ethnicity, n (%)				
White	41,849 (94.7)	49,074 (95.4)	37,802 (95.8)	49,234 (95.4)
Mixed	292 (0.7)	313 (0.6)	221 (0.6)	300 (0.6)
Asian	682 (1.5)	700 (1.4)	505 (1.3)	819 (1.6)
Black	719 (1.6)	624 (1.2)	418 (1.1)	548 (1.1)
Chinese	143 (0.3)	160 (0.3)	108 (0.3)	132 (0.3)
Others	338 (0.8)	378 (0.7)	270 (0.7)	400 (0.8)
Unknown	179 (0.4)	181 (0.4)	122 (0.3)	164 (0.3)
Education, n (%)				
College or university	16,563 (37.5)	21,413 (41.6)	17,461 (44.3)	24,311 (47.1)
Vocational	4894 (11.1)	5309 (10.3)	3996 (10.1)	5176 (10.0)
Upper secondary	5655 (12.8)	6826 (13.3)	5157 (13.1)	6822 (13.2)
Lower secondary	12,235 (27.7)	13,175 (25.6)	9679 (24.5)	11,560 (22.4)
Others	4576 (10.4)	4436 (8.6)	2980 (7.6)	3543 (6.9)
Unknown	279 (0.6)	271 (0.5)	173 (0.4)	185 (0.4)
Townsend deprivation index, median (IQR)	−2.1 (− 3.6 to 0.5)	−2.3 (− 3.7 to 0.1)	−2.4 (− 3.8 to − 0.2)	−2.4 (− 3.8 to − 0.1)
Body mass index, n (%)				
<18.5	181 (0.4)	253 (0.5)	217 (0.6)	350 (0.7)
18.5 ~ 24.9	13,621 (30.8)	18,436 (35.9)	15,203 (38.5)	21,353 (41.4)
25 ~ 39.9	19,070 (43.1)	21,545 (41.9)	16,202 (41.1)	20,674 (40.1)
≥30	11,191 (25.3)	11,046 (21.5)	7732 (19.6)	9089 (17.6)
Unknown	139 (0.3)	150 (0.3)	92 (0.2)	131 (0.3)
Alcohol consumption, n (%)				
Daily or almost daily	11,500 (26.0)	12,175 (23.7)	8539 (21.7)	10,183 (19.7)
3 or 4 times a week	11,026 (24.9)	13,089 (25.5)	9917 (25.1)	12,950 (25.1)
1 or 2 times a week	10,620 (24.0)	12,738 (24.8)	10,142 (25.7)	13,155 (25.5)
1 to 3 times a month	4548 (10.3)	5554 (10.8)	4510 (11.4)	6000 (11.6)
Special occasions only	4015 (9.1)	4880 (9.5)	3909 (9.9)	5444 (10.6)
Never	2440 (5.5)	2949 (5.7)	2393 (6.1)	3840 (7.4)
Unknown	53 (0.1)	45 (0.1)	36 (0.1)	25 (0.1)
Smoking status, n (%)				
Never	23,333 (52.8)	29,102 (56.6)	23,044 (58.4)	30,782 (59.7)
Former smokers	15,971 (36.1)	18,047 (35.1)	13,635 (34.6)	17,698 (34.3)
Current smokers	4771 (10.8)	4150 (8.1)	2653 (6.7)	3003 (5.8)
Unknown	127 (0.3)	131 (0.3)	114 (0.3)	114 (0.2)
Physical activity, n (%)				
Low	8112 (18.4)	8462 (16.5)	5886 (14.9)	6661 (12.9)
Moderate	15,535 (35.2)	18,639 (36.2)	14,291 (36.2)	18,523 (35.9)
High	13,535 (30.6)	16,445 (32.0)	13,221 (33.5)	19,120 (37.1)
Unknown	7020 (15.9)	7884 (15.3)	6048 (15.3)	7293 (14.1)
Energy intake, mean (SD), kcal/d	1947.7 (573.7)	1996.1 (535.5)	2047.7 (518.6)	2169.5 (529.1)

Data were expressed as mean (SD) or number of participants (proportion). Nonparametric tests were used for continuous variables and chi-square tests were used for categorical variables. All tests had *P* values less than 0.001

*CRC* colorectal cancer, *IQR* inter-quartile range, *SD* standard deviation

### Association between PDIs and CRC incidence

**Fig. 1:**
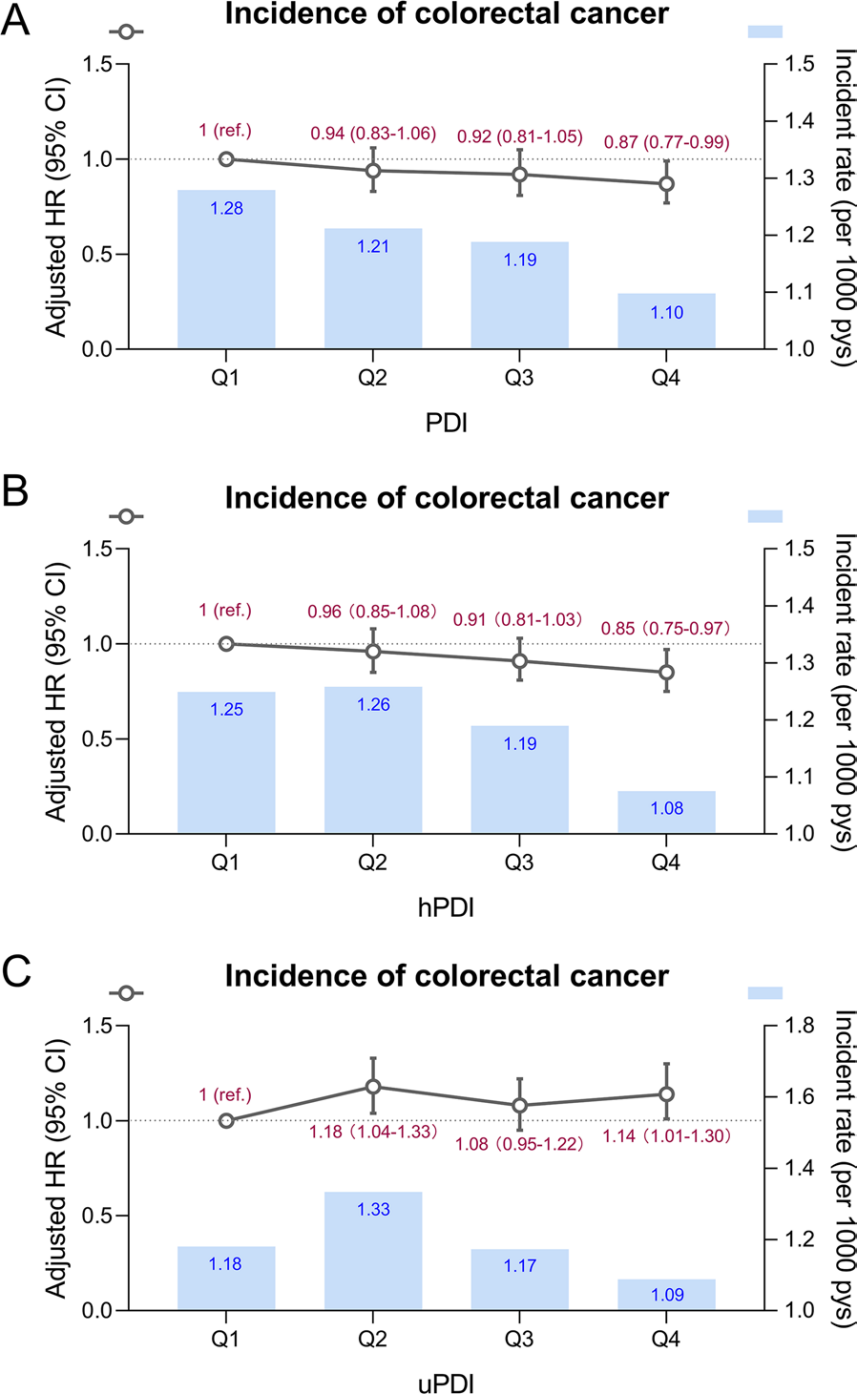
Associations of PDI, hPDI, and uPDI with risk of CRC incidence. The models adjusted for age (continuous), sex (female, male), ethnicity (White, mixed, Asian, Black, Chinese, others, or unknown), education (college or university, vocational qualification, upper secondary, lower secondary, others, or unknown), Townsend deprivation index (in quintiles), body mass index (< 18.5, 18.5–24.9, 25-29.9, or ≥ 30 kg/m^2^), alcohol frequency (daily or almost daily, 3 or 4 times a week, 1 or 2 times a week, 1 to 3 times a month, special occasions only, never, or unknown), smoking status (never, former, current, or unknown), physical activity (low, moderate, high, or unknown), total energy intake (continuous), polygenic risk score for CRC (continuous), first 10 principal components of ancestry (in Units, continuous), and genotype measurement batch (continuous). *CI* confidence interval, *CRC* colorectal cancer, *hPDI* healthful plant-based diet index, *HR* hazard ratio, *PDI* plant-based diet index, *uPDI* unhealthful plant-based diet index

During a median of 9.5 years of follow-up (interquartile range [IQR], 9.4–10.3 years), 2163 CRC cases were documented. We did not observe significant departures from linearity when the non-linearity of PDIs with the incidence of CRC was tested (*P*_non−linearity_ >0.05; Additional file 1: Fig. S2). Compared to the lowest quartile, multivariable-adjusted HRs of CRC incidence in the highest quartile were 0.87 (95% CI, 0.77–0.99) and 0.85 (95% CI, 0.75–0.97) for PDI and hPDI, respectively, and that in the second and highest quartile were 1.18 (95% CI, 1.04–1.33) and 1.14 (95% CI, 1.01–1.30) for uPDI, respectively (Fig. 1 and Additional file 1: Table S7). Additionally, per 10-score increments of PDI and hPDI were associated with 12% and 9% lower risks of CRC incidence, respectively.

Concerning different anatomical subsites of CRC, the Q4 level of hPDI (HR: 0.77 [95% CI, 0.60–0.98]) and uPDI (HR: 1.30 [95% CI, 1.02–1.65]) were observed to be negatively and positively associated with risk of distal colon cancer, respectively (Table 2). Higher PDI (*P*_trend_ = 0.0093) and hPDI (*P*_trend_ = 0.0330) were associated with a reduced risk of rectal cancer. None of the three PDIs were associated with the risk of proximal colon cancer.

**Table 2:** Association between plant-based diet indices and risk of CRC incidence classified by anatomical subsites

Plant-based diet indices	Proximal colon cancer			Distal colon cancer			Rectal cancer		
	Cases/ person-years	Incident rate per 1000 pys	HR (95% CI)	Cases/ person-years	Incident rate per 1000 pys	HR (95% CI)	Cases/ person-years	Incident rate per 1000 pys	HR (95% CI)
*PDI*									
Q1 (24–46)	195/435,153	0.45	1.00 (ref.)	157/435,237	0.36	1.00 (ref.)	277/434,817	0.64	1.00 (ref.)
Q2 (47–49)	238/500,520	0.48	1.04 (0.86–1.26)	181/500,572	0.36	1.02 (0.82–1.26)	265/500,355	0.53	**0.84 (0.71–0.99)**
Q3 (50–53)	190/383,024	0.50	1.08 (0.88–1.33)	130/383,131	0.34	0.97 (0.77–1.23)	215/382,902	0.56	0.89 (0.75–1.07)
Q4 (54–77)	237/501,317	0.47	1.05 (0.86–1.27)	164/501,442	0.33	0.96 (0.76–1.20)	235/501,266	0.47	**0.76 (0.64–0.91)**
*P* trend *			0.6154			0.6066			**0.0093**
Per 10 increases			0.97 (0.85–1.10)			0.91 (0.78–1.05)			**0.93 (0.88–0.98)**
*hPDI*									
Q1 (29–50)	202/439,973	0.46	1.00 (ref.)	164/440,025	0.37	1.00 (ref.)	260/439,722	0.59	1.00 (ref.)
Q2 (51–54)	215/452,866	0.47	0.97 (0.80–1.18)	165/452,851	0.36	0.97 (0.78–1.20)	267/452,585	0.59	0.98 (0.83–1.17)
Q3 (55–58)	214/460,191	0.47	0.93 (0.76–1.14)	176/460,322	0.38	1.03 (0.82–1.28)	242/460,146	0.53	0.88 (0.73–1.05)
Q4 (59–82)	229/466,982	0.49	1.01 (0.82–1.23)	127/467,185	0.27	**0.77 (0.60–0.98)**	223/466,888	0.48	0.84 (0.69–1.01)
*P* trend *			0.9655			0.0730			**0.0330**
Per 10 increases			1.00 (0.88–1.13)			0.91 (0.79–1.06)			**0.94 (0.88–0.99)**
*uPDI*									
Q1 (28–51)	199/406,269	0.49	1.00 (ref.)	123/406,531	0.30	1.00 (ref.)	224/406,159	0.55	1.00 (ref.)
Q2 (52–55)	238/455,279	0.52	1.13 (0.93–1.36)	190/455,222	0.42	**1.42 (1.13–1.78)**	276/455,052	0.61	1.15 (0.96–1.37)
Q3 (56–58)	213/472,971	0.45	1.03 (0.85–1.26)	158/473,084	0.33	1.18 (0.93–1.50)	264/472,754	0.59	1.11 (0.92–1.33)
Q4 (59–79)	210/485,493	0.43	1.16 (0.95–1.42)	161/485,545	0.33	**1.30 (1.02–1.65)**	228/485,376	0.47	1.04 (0.86–1.26)
*P* trend *			0.2813			0.1653			0.7960
Per 10 increases			1.06 (0.94–1.20)			1.07 (0.93–1.24)			1.01 (0.95–1.07)

The bold values indicate that the test is significant (*P* < 0.05)

*Linear trend was tested by treating the plant-based diet index category as a continuous variable

The models adjusted for age (continuous) and sex (female, male), ethnicity (White, mixed, Asian, Black, Chinese, others, or unknown), education (college or university, vocational qualification, upper secondary, lower secondary, others, or unknown), Townsend deprivation index (in quintiles), body mass index (< 18.5, 18.5–24.9, 25–29.9, or ≥ 30 kg/m^2^), alcohol frequency (daily or almost daily, 3 or 4 times a week, 1 or 2 times a week, 1 to 3 times a month, special occasions only, never, or unknown), smoking status (never, former, current, or unknown), physical activity (low, moderate, high, or unknown), total energy intake (continuous), polygenic risk score for CRC (continuous), first 10 principal components of ancestry (in Units, continuous), and genotype measurement batch (continuous)

*CI* confidence interval, *CRC* colorectal cancer, *hPDI* healthful plant-based diet index, *HR* hazard ratio, *PDI* overall plant-based diet index, *uPDI* unhealthful plant-based diet index

### The modification by genetic risk on the PDIs-CRC associations

There existed a non-linear relationship between PRS and CRC incidence (*P*_non−linearity_ >0.05; Additional file 1: Fig. S3), and per SD increment of PRS accounted for a 45% increased risk of CRC incidence.

In stratified analyses by genetic risk, we observed a reduced risk of CRC incidence conferred by hPDI in subjects with low genetic risk and by PDI in those with intermediate and high genetic risk (Additional file 1: Table S8). In addition, no interaction between PDIs and PRS for CRC incidence was observed (*P*_interaction_ >0.05).

**Fig. 2:**
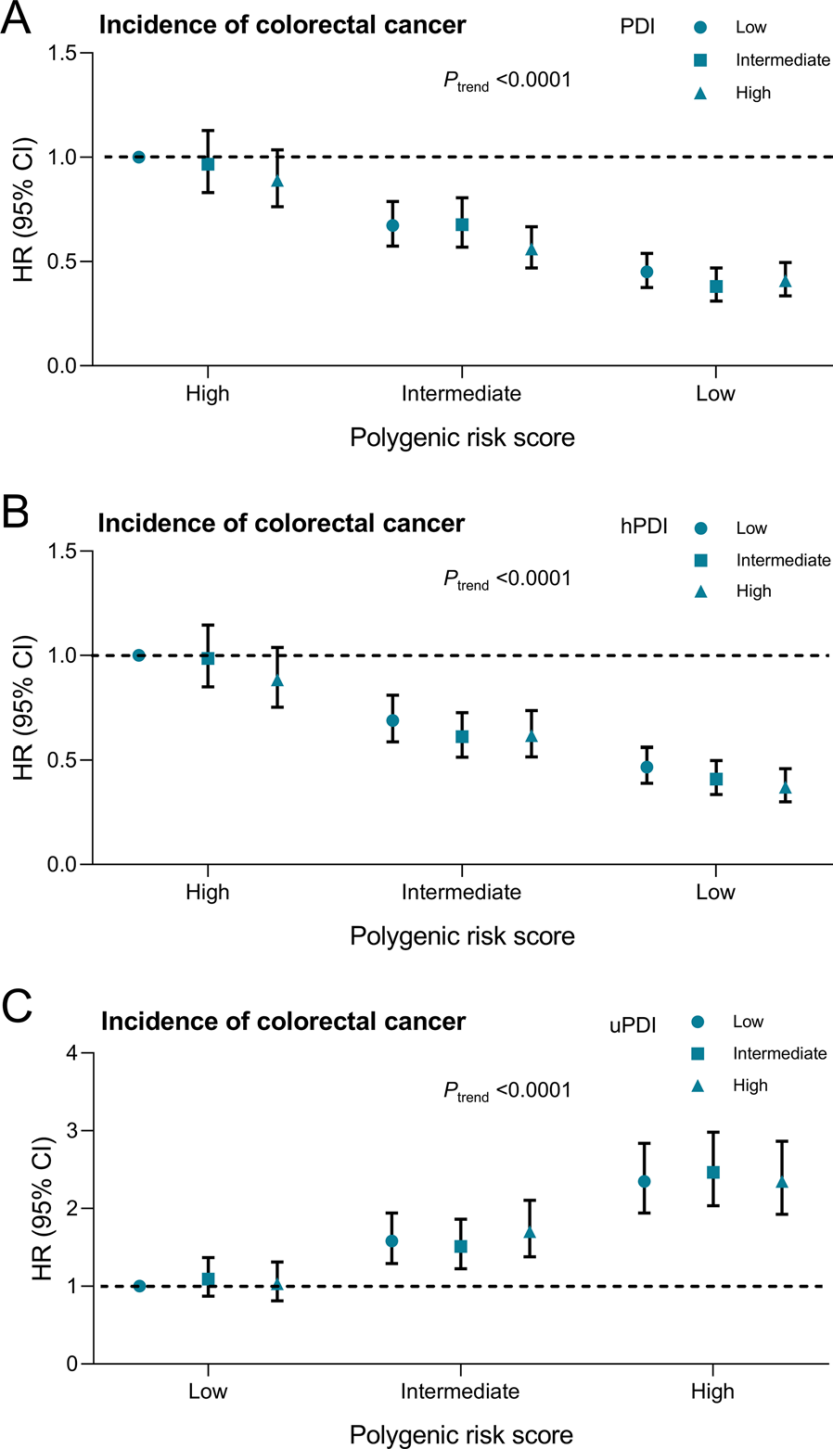
Joint Associations of PDI, hPDI, and uPDI and PRS with risk of CRC incidence. The models adjusted for age (continuous), sex (female, male), ethnicity (White, mixed, Asian, Black, Chinese, others, or unknown), education (college or university, vocational qualification, upper secondary, lower secondary, others, or unknown), Townsend deprivation index (in quintiles), body mass index (< 18.5, 18.5–24.9, 25-29.9, or ≥ 30 kg/m^2^), alcohol frequency (daily or almost daily, 3 or 4 times a week, 1 or 2 times a week, 1 to 3 times a month, special occasions only, never, or unknown), smoking status (never, former, current, or unknown), physical activity (low, moderate, high, or unknown), total energy intake (continuous), first 10 principal components of ancestry (in Units, continuous), and genotype measurement batch (continuous). *CI* confidence interval, *CRC* colorectal cancer, *hPDI* healthful plant-based diet index, *HR* hazard ratio, *PDI* plant-based diet index, *uPDI* unhealthful plant-based diet index

The joint analysis showed a risk gradient with increasing genetic risk and decreasing PDIs quality (Fig. 2). Compared with individuals at the highest PRS and lowest PDI/hPDI category, the multivariable-adjusted HRs for CRC risk were 0.41 (95% CI, 0.34–0.50) among those at the lowest PRS and highest PDI category, and 0.37 (95% CI, 0.30–0.46) among those at the lowest PRS and highest hPDI category. Compared to those with the lowest PRS and uPDI, the multivariable-adjusted HR for CRC risk was 2.35 (95% CI, 1.92–2.87) in the highest PRS and uPDI.

### Association between PDIs and CRC incidence stratified by subgroups

In the fully adjusted models, a significant association of the Q2 (HR: 1.37 [95% CI, 1.14–1.65]) and Q4 (HR: 1.29 [95% CI, 1.05–1.58]) levels of uPDI (*P*_trend_ =0.0472) with an increased risk of CRC incidence was observed in females, whereas a reduced risk of CRC incidence conferred by higher PDI (HR_Q4_: 0.78 [95% CI, 0.66–0.92], *P*_trend_ =0.0028) and hPDI (HR_Q4_: 0.79 [95% CI, 0.67–0.95], *P*_trend_ =0.0069) was reported only in males (Additional file 1: Table S9).

We observed an inverse association of PDI with CRC incidence in participants who had lower Townsend deprivation index and normal BMI, drank alcohol frequently and had moderate physical activity (Additional file 1: Table S10). The negative association of hPDI with CRC incidence was revealed in older participants, who were less deprived and overweight, drank less alcohol, and never smoked (Additional file 1: Table S11). Meanwhile, we observed an interaction between hPDI and age (*P*_interaction_ =0.0238). For uPDI, the positive association was restricted to older adults, non-smokers, and those with normal BMI and less alcohol intake (Additional file 1: Table S12).

### Association between PDIs and CRC mortality

**Fig. 3:**
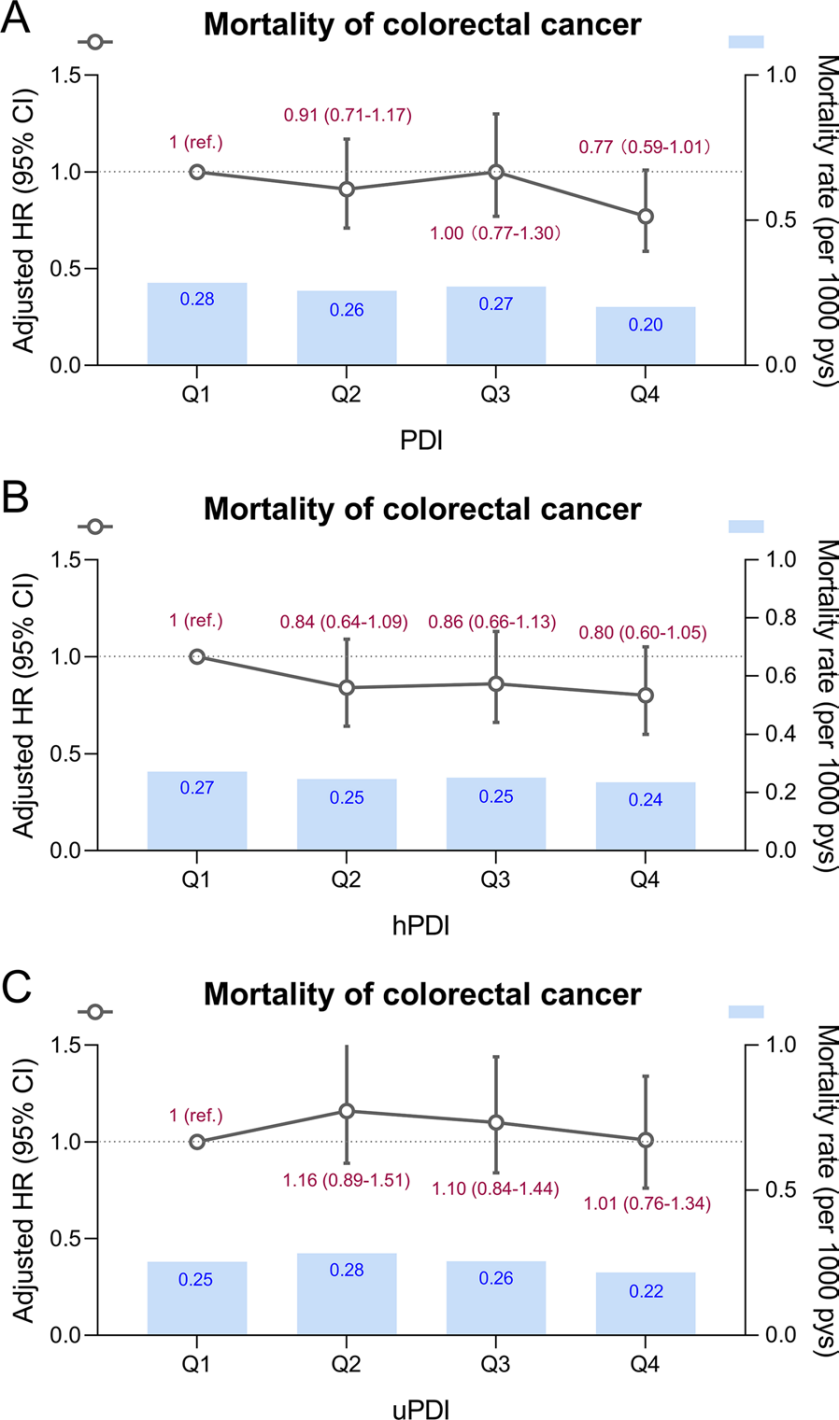
Associations of PDI, hPDI, and uPDI with risk of CRC mortality. The models adjusted for age (continuous), sex (female, male), ethnicity (White, mixed, Asian, Black, Chinese, others, or unknown), education (college or university, vocational qualification, upper secondary, lower secondary, others, or unknown), Townsend deprivation index (in quintiles), body mass index (< 18.5, 18.5–24.9, 25-29.9, or ≥ 30 kg/m2), alcohol frequency (daily or almost daily, 3 or 4 times a week, 1 or 2 times a week, 1 to 3 times a month, special occasions only, never, or unknown), smoking status (never, former, current, or unknown), physical activity (low, moderate, high, or unknown), total energy intake (continuous), polygenic risk score for CRC (continuous), first 10 principal components of ancestry (in Units, continuous), and genotype measurement batch (continuous). *CI* confidence interval, *CRC* colorectal cancer, *hPDI* healthful plant-based diet index, *HR* hazard ratio, *PDI* plant-based diet index, *uPDI* unhealthful plant-based diet index

A total of 466 CRC deaths occurred after a median of 9.9 years of follow-up (IQR, 9.5–10.4 years). We did not observe a non-linear relationship between PDIs and CRC mortality (*P*_non−linearity_ >0.05; Additional file 1: Fig. S4). As presented in Fig. 3 and Additional file 1: Table S13, the age-sex adjusted model showed a decreased risk of CRC mortality with the highest PDI (HR: 0.71 [95% CI, 0.55–0.92]), which was eliminated after additional adjustment for all covariates. However, the inverse association of PDI with CRC mortality was still present among males (Additional file 1: Table S14). Interestingly, hPDI showed a protective tendency in the male population (*P*_trend_ =0.0388).

Additionally, a null association between PDIs and CRC mortality was independent of genetic risk, and no significant interaction was found (*P*_interaction_ >0.05; Additional file 1: Table S15).

### Secondary analyses

The inverse association of hPDI with CRC risk disappeared when further excluding participants with less than two years of follow-up. The PDIs-CRC associations remained largely unchanged when using sub-distribution hazard models for competing risk (Additional file 1: Table S16). In addition, we observed a negative association between the intake of healthy food groups and CRC risk in males (Additional file 1: Table S17).

We further modified the PDI and hPDI by firstly assigning a positive score to dairy products (as beneficial components, HR: 0.96 [95% CI, 0.94–0.99]) and by secondly assigning positive scores to both dairy products and seafood (as potential beneficial components, HR: 0.97 [95% CI, 0.92–1.02]). We did not observe any non-linearity in the association of the modified PDI/hPDI and CRC risk (All *P*_non−linearity_ >0.05; Additional file 1: Fig. S5). The results of both sensitivity analyses remained stable (Additional file 1: Table S18).

## Discussion

In this large prospective study, we found that independent of genetic predisposition, greater adherence to PDI and hPDI was associated with a lower risk of CRC, predominantly distal CRC. The inverse association of PDI and hPDI with the risk of CRC incidence and mortality was more pronounced in males, but uPDI was positively associated with CRC incidence risk only among females. In the joint analysis, we observed a gradually decreased CRC risk ascribed to higher PDIs quality combined with lower genetic risk.

Over the years, following a plant-based diet has become increasingly popular, and studies have linked vegetarian diets to CRC risk. A meta-analysis of 3,059,009 subjects demonstrated that diets rich in plant-based food were associated with a lower risk of digestive system cancers, especially CRC [33]. Subsequently, two large-scale cohort studies from the UK Biobank concluded that low meat-eaters, even vegetarians, had a decreased risk of CRC compared with regular meat-eaters [34, 35]. However, adherence to a strict vegetarian or vegan diet has been challenging for a long time. Furthermore, these diets did not distinguish between healthier and lower-quality plant-based foods [36]. Therefore, Satija et al. proposed the PDIs considering the quality of plant-based foods [25]. However, previous evidence on associations between plant-based diets and CRC risk has been inconclusive. A case-control study in China observed an inverse association of hPDI but a positive association of uPDI with CRC risk [14]. A recent study in the Nurses’ Health Study (NHS) and the Health Professionals Follow-up Study (HPFS) obtained similar results and found a negative association of hPDI, especially with *KRAS*‐wildtype CRC [15]. However, a prospective cohort of women aged 26–45 years in the NHSII and another study of subjects in the HPFS, NHS and NHSII found that the three PDIs were not associated with CRC risk [16, 17]. The latest study from the UK explored the associations of hPDI and uPDI with risk of mortality and major chronic diseases and only found a positive association of Q2 and Q3 levels of uPDI with CRC risk [18]. Herein, we comprehensively and more deeply examined the associations between three PDIs and CRC-specific outcomes using a larger-scale sample size and found that the inverse associations of PDI and hPDI but the positive association of uPDI with CRC risk remained significant in the final model and sensitivity analyses. These findings supported evidence-based preventive interventions and highlighted the potential importance of the quality of plant-based foods for CRC prevention.

The hypothesis of gene-diet interactions in the etiology of CRC has long been supported [37]. A Danish nested study of 1038 cases and 1857 controls showed that *CCAT2* rs6983267 T-allele carriers had a lower relative risk of CRC by red and processed meat intake compared to GG homozygotes [38]. Another case-control study of 9243 participants observed that red and processed meat intake increased CRC risk regardless of PRS levels [39]. The interplay between the overall genetic risk and the whole diet quality (e.g., PDIs) for CRC has not been reported. In the present study, we found that both PRS and PDIs could independently predict CRC risk. However, the inverse associations of PDI and hPDI and a positive association of uPDI with CRC risk were independent of genetic predisposition without any interactions, which signified that people with different genetic risks should all value the quality of plant foods.

Studies have explored the specific associations of plant-based diets and even vegetarianism with the anatomical subsites of CRC; however, these varied depending on the study design [33]. A previous meta-analysis of cohort studies reported no significant association between vegetarianism and colon and rectal cancer risk [40]. In contrast, our stratified analysis by CRC localization found that the effect of PDIs was more concentrated in the distal CRC, which was consistent with the results from the Multiethnic Cohort Study [19]. This might be ascribed to different distributions of the intestinal microbiome in various parts of the gut [41], and compared with the colon, the rectum is more susceptible to genotoxic and cytotoxic damage due to its longer transit time and the large accumulation of feces prior to defecation [42]. The present findings emphasized the role of plant-rich diets in the prevention of distal CRC.

Sex differences were observed in our results. Generally, the females consume more plant foods and fewer animal foods than the males [14]. In our study population, the females ate more healthy plant foods and less unhealthy plant foods, so there may be no further benefits from healthy plant foods, but they may suffer the harms of unhealthy plant foods. Besides, the males had a higher risk of CRC than the females [43], suggesting that a plant-based diet may offer more benefits for the males than the females in reducing risk.

The protective association of a high-quality plant-based diet with CRC could be partly attributable to food components and nutrients with antioxidant and anti-inflammatory properties. Nutrients abundant in healthy plant foods (e.g., polyphenols, such as proanthocyanidins and anthocyanin 3-glucosides in fruits and vegetables) were reported to act as antioxidants to inhibit the production of pro-inflammatory cytokines [44, 45] and have protective activities against CRC [46]. High levels of antioxidant micronutrients, such as vitamin E, vitamin C, carotenoids, and phytochemicals present in healthy plant-based diets, were related to lower levels of inflammation, while low-quality plant-based foods and meat could be proinflammatory [36, 47]. Furthermore, dietary fiber from whole grains, fruits, and vegetables processed protective activity on CRC by regulating prebiotic microbiota and fermentation rate [7]. These features of healthy plant-based diets might conduce to the prevention of CRC and should be taken into account in dietary recommendations for the general population.

The prospective study design and the large sample size were the two main strengths of this study. To our knowledge, this was the first longitudinal study to comprehensively investigate the association of plant-based diets with risks of CRC incidence and mortality considering genetic predisposition in the general population. Several limitations should be mentioned. First, due to a 5.5% participation rate in the UK Biobank, the recruitment was influenced by selection bias [48]. Studies have demonstrated that the lack of representativeness in the UK Biobank does not materially affect the associations between diets and health outcomes [49], but rather distorts genetic associations and downstream analyses [50]. Therefore, with respect to the analysis of genetic data, our study population may not be completely representative of the UK population. Second, the dietary assessment was based on 24-hour recall, which might be subjected to measurement error and lead to misclassification. Third, only 17 food groups were used to construct the PDIs due to the unavailability of vegetable oils in the current study, which was included in the original paper describing the PDIs by Satija et al. [25]. Fourth, the PDIs treat all animal-based foods equally without discrimination by assigning opposite scores, which may ignore benefits from some food components, such as dairy products and seafood. However, the results of our sensitivity analyses were stable by considering dairy products and seafood as healthful food groups. Fifth, we could not further subdivide meat into red and white meats, the latter of which may be associated with a reduced CRC risk [51]. Sixth, even though we had controlled the majority of confounders, the residual confounding from unmeasured or unknown factors might remain. Finally, our analyses were conducted among Europeans, limiting the extrapolation of our findings to other ethnic groups.

## Conclusions

Our results suggested that adherence to higher-quality plant-based diets was associated with a lower risk of CRC incidence, particularly in distal CRC (distal colon and rectal cancer). Increased quality of plant-based diets combined with decreased genetic risk may have more benefits against CRC. These findings provided suggestions for future research on the importance of food quality when adhering to a plant-based dietary pattern for the prevention of CRC in the general population with different genetic predispositions.

### Supplementary information

**Additional file 1: Figure S1.** Flow chart of the study design. **Figure S2.** Restricted cubic splines for plant-based diet indices and risk of CRC incidence. **Figure S3.** Restricted cubic spline for polygenic risk score and risk of CRC incidence. **Figure S4.** Restricted cubic splines for plant-based diet indices and risk of CRC mortality. **Figure S5.** Restricted cubic splines for the modified PDI/hPDI and risks of CRC incidence and mortality. **Table S1.** Definition of CRC in the UK Biobank Study. **Table S2.** Examples of food items constituting the 17 food groups in UK Biobank study. **Table S3.** Scores of food items of 186675 participants by plant-based diet indices groups. **Table S4.** List of 95 SNPs included in the polygenic risk score for CRC. **Table S5.** Baseline characteristics of 186675 participants by hPDI groups. **Table S6.** Baseline characteristics of 186675 participants by uPDI groups. **Table S7.** Association between plant-based diet indices and risk of CRC incidence. **Table S8.** Association between plant-based diet indices and risk of CRC incidence according to categories of genetic risk. **Table S9.** Subgroup analysis for the association between plant-based diet indices and risk of CRC incidence by sex. **Table S10.** Subgroup analysis for the association between PDI and risk of CRC incidence. **Table S11.** Subgroup analysis for the association between hPDI and risk of CRC incidence. **Table S12.** Subgroup analysis for the association between uPDI and risk of CRC incidence. **Table S13.** Association between plant-based diet indices and risk of CRC mortality. **Table S14.** Subgroup analysis for the association between plant-based diet indices and risk of CRC mortality by sex. **Table S15.** Association between plant-based diet indices and risk of CRC mortality according to categories of genetic risk. **Table S16.** Sensitivity analyses for the association between plant-based diet indices and risks of CRC incidence and mortality. **Table S17.** Association between 3 food categories and risks of CRC incidence and mortality. **Table S18.** Association between the modified PDI/hPDI and risks of CRC incidence and mortality.

## Data Availability

Data from the UK Biobank are available on application at www.ukbiobank.ac.uk/register-apply.

## Abbreviations

BMI: Body mass index
CI: Confidence interval
CRC: Colorectal cancer
hPDI: Healthful plant-based diet index
HPFS: Health Professionals Follow-up Study
HR: Hazard ratio
ICD: International Classification of Diseases
IQR: Interquartile range
NHS: Nurses’ Health Study
PDIs: Plant-based diet indices
PDI: Overall plant-based diet index
PRS: Polygenic risk score
RCS: Restricted cubic splines
SD: Standard deviation
SNP: Single-nucleotide polymorphism
TEI: Total energy intake
uPDI: Unhealthful plant-based diet index
