# Supplementary material for: Plant-based dietary patterns, genetic predisposition and risk of colorectal cancer: a prospective study from the UK Biobank
Source: J Transl Med. 2023 Sep 27;21:669. doi: 10.1186/s12967-023-04522-8 (PMC10536761; doi:10.1186/s12967-023-04522-8)
Supplement: Supplementary file 1 — Additional file 1: Figure S1. Flow chart of the study design. Figure S2. Restricted cubic splines for plant-based diet indices and risk of CRC incidence. Figure S3. Restricted cubic spline for polygenic risk score and risk of CRC incidence. Figure S4. Restricted cubic splines for plant-based diet indices and risk of CRC mortality. Figure S5. Restricted cubic splines for the modified PDI/hPDI and risks of CRC incidence and mortality. Table S1. Definition of CRC in the UK Biobank Study. Table S2. Examples of food items constituting the 17 food groups in UK Biobank study. Table S3. Scores of food items of 186675 participants by plant-based diet indices groups. Table S4. List of 95 SNPs included in the polygenic risk score for CRC. Table S5. Baseline characteristics of 186675 participants by hPDI groups. Table S6. Baseline characteristics of 186675 participants by uPDI groups. Table S7. Association between plant-based diet indices and risk of CRC incidence. Table S8. Association between plant-based diet indices and risk of CRC incidence according to categories of genetic risk. Table S9. Subgroup analysis for the association between plant-based diet indices and risk of CRC incidence by sex. Table S10. Subgroup analysis for the association between PDI and risk of CRC incidence. Table S11. Subgroup analysis for the association between hPDI and risk of CRC incidence. Table S12. Subgroup analysis for the association between uPDI and risk of CRC incidence. Table S13. Association between plant-based diet indices and risk of CRC mortality. Table S14. Subgroup analysis for the association between plant-based diet indices and risk of CRC mortality by sex. Table S15. Association between plant-based diet indices and risk of CRC mortality according to categories of genetic risk. Table S16. Sensitivity analyses for the association between plant-based diet indices and risks of CRC incidence and mortality. Table S17. Association between 3 food categories and risks of CRC incidence and [file 12967_2023_4522_MOESM1_ESM.doc]

**Plant-based Dietary Patterns, Genetic Predisposition and Risk of Colorectal Cancer:**

**A Prospective Study from the UK Biobank**

Fubin Liu, MD ^1^, Yanling Lv, MD ^2^, Yu Peng, MD ^1^, Yating Qiao, MD ^1^, Peng Wang, MD ^1^, Changyu Si, MD ^1^, Xixuan Wang, MD ^1^, Jianxiao Gong, MD ^1^, Huijun Zhou, MD ^1^, Ming Zhang, PhD ^3^, Liangkai Chen, PhD ^2,*^, Fangfang Song, PhD ^1,*^

^1^ Department of Epidemiology and Biostatistics, Key Laboratory of Molecular Cancer Epidemiology, Tianjin, National Clinical Research Center for Cancer, Tianjin’s Clinical Research Center for Cancer, Tianjin Medical University Cancer Institute and Hospital, Tianjin, 300060, China.

^2^ Department of Nutrition and Food Hygiene, Hubei Key Laboratory of Food Nutrition and Safety, Ministry of Education Key Lab of Environment and Health, School of Public Health, Tongji Medical College, Huazhong University of Science and Technology, Wuhan, 430030, China.

^3^ Comprehensive Management Department of Occupational Health, Shenzhen Prevention and Treatment Center for Occupational Diseases, Shenzhen, 518020, China.

**Additional file 1**

**Figure S1.** Flow chart of the study design

**Figure S2.** Restricted cubic splines for plant-based diet indices and risk of CRC incidence

**Figure S3.** Restricted cubic spline for polygenic risk score and risk of CRC incidence

**Figure S4.** Restricted cubic splines for plant-based diet indices and risk of CRC mortality

**Figure S5.** Restricted cubic splines for the modified PDI/hPDI and risks of CRC incidence and mortality

**Table S1.** Definition of CRC in the UK Biobank Study

**Table S2.** Examples of food items constituting the 17 food groups in UK Biobank study

**Table S3.** Scores of food items of 186675 participants by plant-based diet indices groups

**Table S4.** List of 95 SNPs included in the polygenic risk score for CRC

**Table S5.** Baseline characteristics of 186675 participants by hPDI groups

**Table S6.** Baseline characteristics of 186675 participants by uPDI groups

**Table S7.** Association between plant-based diet indices and risk of CRC incidence

**Table S8.** Association between plant-based diet indices and risk of CRC incidence according to categories of genetic risk

**Table S9.** Subgroup analysis for the association between plant-based diet indices and risk of CRC incidence by sex

**Table S10.** Subgroup analysis for the association between PDI and risk of CRC incidence

**Table S11.** Subgroup analysis for the association between hPDI and risk of CRC incidence

**Table S12.** Subgroup analysis for the association between uPDI and risk of CRC incidence

**Table S13.** Association between plant-based diet indices and risk of CRC mortality

**Table S14.** Subgroup analysis for the association between plant-based diet indices and risk of CRC mortality by sex

**Table S15.** Association between plant-based diet indices and risk of CRC mortality according to categories of genetic risk

**Table S16.** Sensitivity analyses for the association between plant-based diet indices and risks of CRC incidence and mortality

**Table S17.** Association between 3 food categories and risks of CRC incidence and mortality

**Table S18.** Association between the modified PDI/hPDI and risks of CRC incidence and mortality


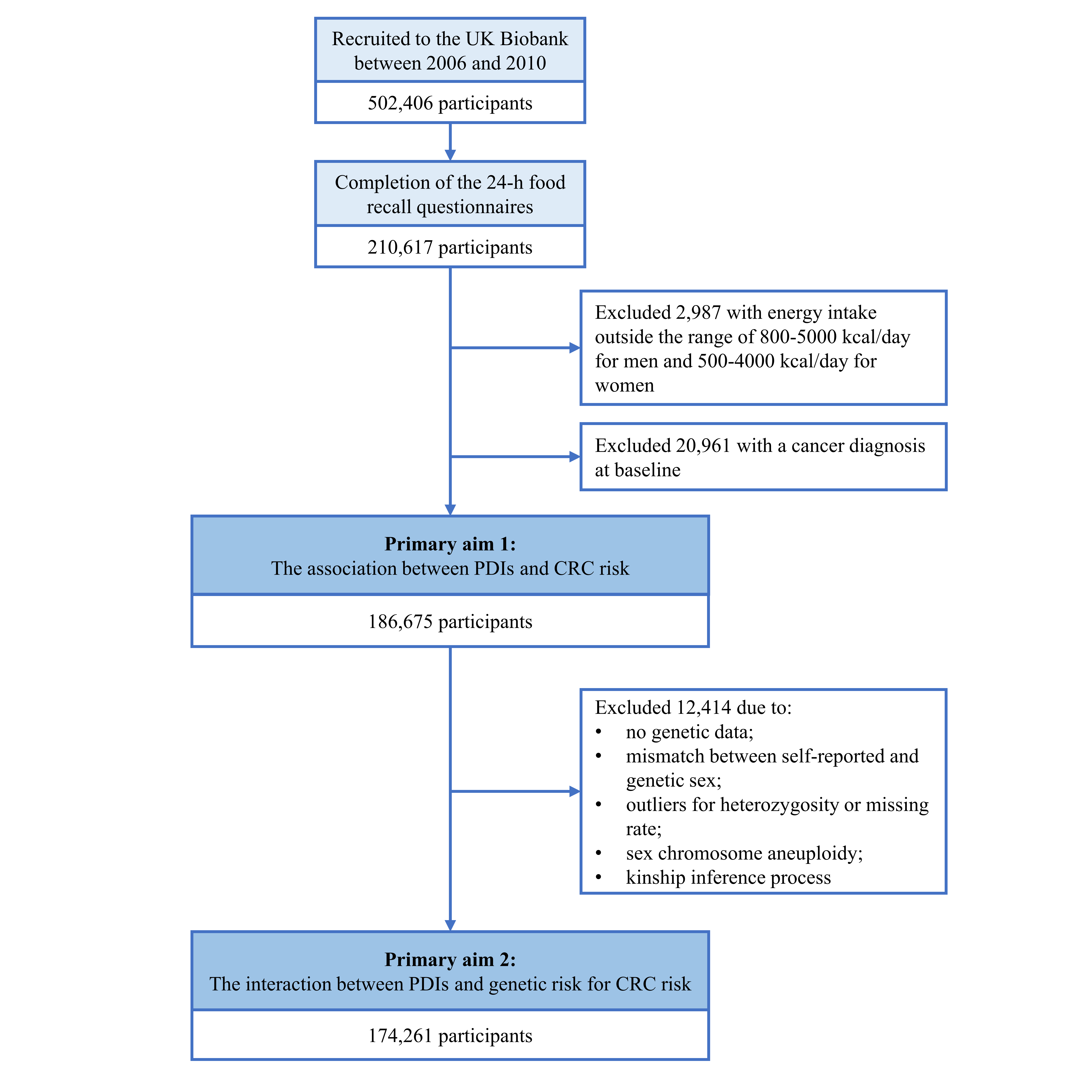


**Figure S1.** Flow chart of the study design

Abbreviations: CRC, colorectal cancer; PDIs, plant-based dietary indices.


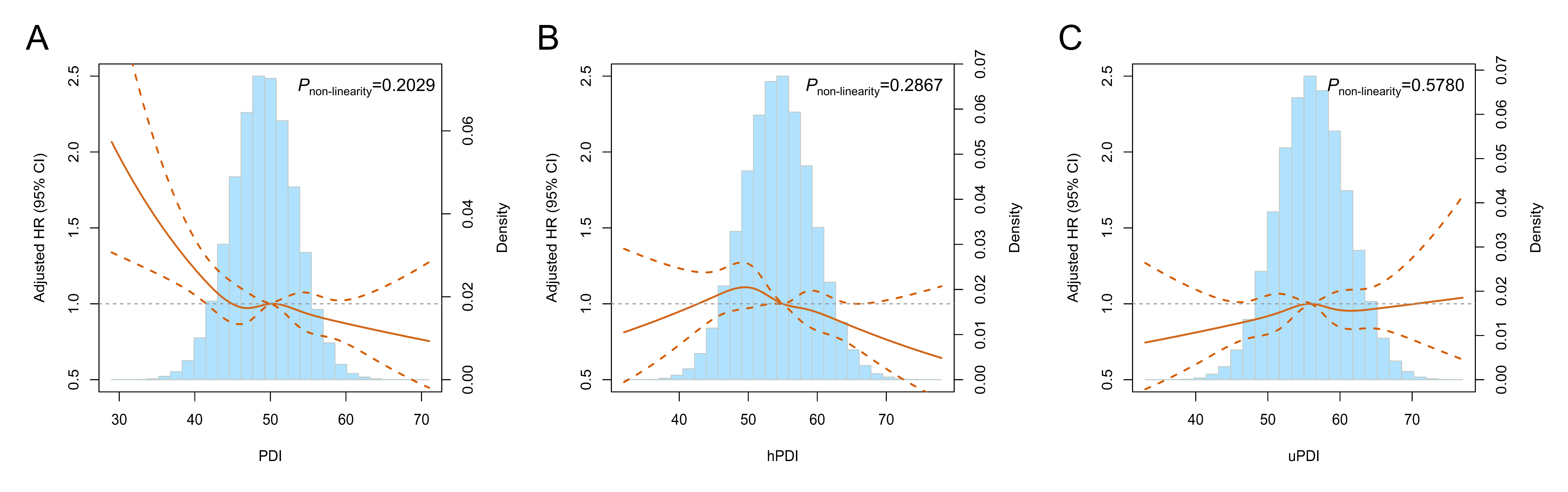


**Figure S2.** Restricted cubic splines for plant-based diet indices and risk of CRC incidence

The models adjusted for age (continuous), sex (female, male), ethnicity (White, mixed, Asian, Black, Chinese, others, or unknown), education (college or university, vocational qualification, upper secondary, lower secondary, others, or unknown), Townsend deprivation index (in quintiles), body mass index (<18.5, 18.5-24.9, 25-29.9, or ≥30 kg/m2), alcohol frequency (daily or almost daily, 3 or 4 times a week, 1 or 2 times a week, 1 to 3 times a month, special occasions only, never, or unknown), smoking status (never, former, current, or unknown), physical activity (low, moderate, high, or unknown), total energy intake (continuous), polygenic risk score for CRC (continuous), first 10 principal components of ancestry (in Units, continuous), and genotype measurement batch (continuous).

Abbreviations: CI, confidence interval; CRC, colorectal cancer; hPDI, healthful plant-based diet index; HR, hazard ratio; PDI, plant-based diet index; uPDI, unhealthful plant-based diet index.


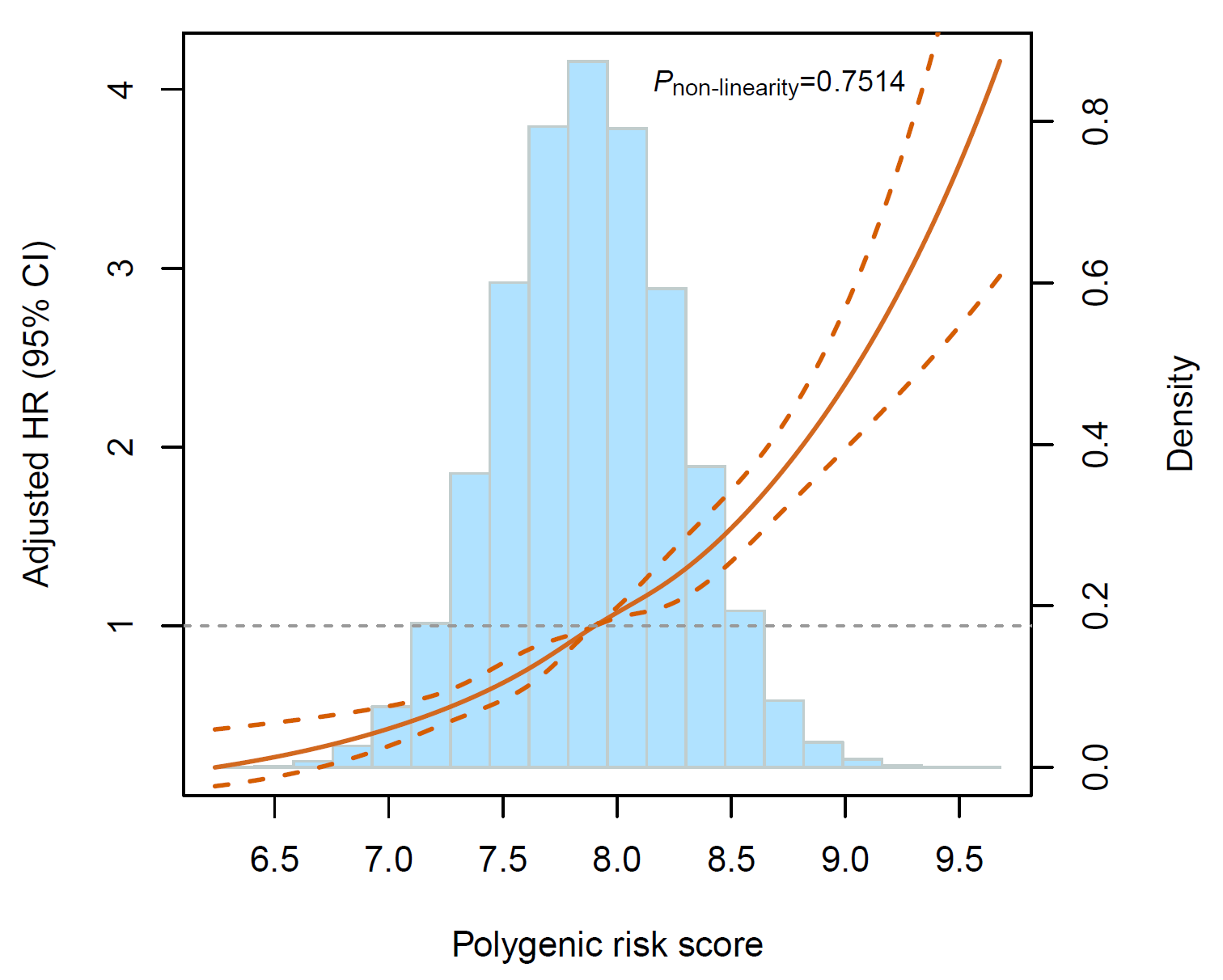


**Figure S3.** Restricted cubic spline for polygenic risk score and risk of CRC incidence

The models adjusted for age (continuous), sex (female, male), ethnicity (White, mixed, Asian, Black, Chinese, others, or unknown), education (college or university, vocational qualification, upper secondary, lower secondary, others, or unknown), Townsend deprivation index (in quintiles), body mass index (<18.5, 18.5-24.9, 25-29.9, or ≥30 kg/m2), alcohol frequency (daily or almost daily, 3 or 4 times a week, 1 or 2 times a week, 1 to 3 times a month, special occasions only, never, or unknown), smoking status (never, former, current, or unknown), physical activity (low, moderate, high, or unknown), total energy intake (continuous), first 10 principal components of ancestry (in Units, continuous), and genotype measurement batch (continuous).

Abbreviations: CI, confidence interval; CRC, colorectal cancer; HR, hazard ratio.

**
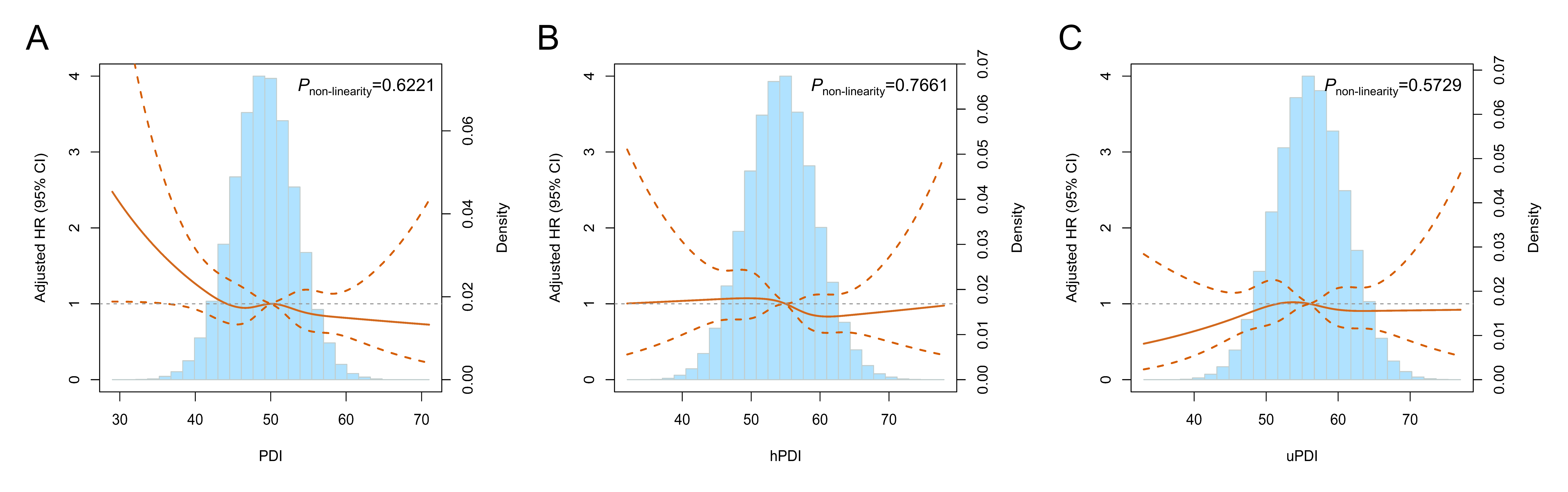
**

**Figure S4.** Restricted cubic splines for plant-based diet indices and risk of CRC mortality

The models adjusted for age (continuous), sex (female, male), ethnicity (White, mixed, Asian, Black, Chinese, others, or unknown), education (college or university, vocational qualification, upper secondary, lower secondary, others, or unknown), Townsend deprivation index (in quintiles), body mass index (<18.5, 18.5-24.9, 25-29.9, or ≥30 kg/m2), alcohol frequency (daily or almost daily, 3 or 4 times a week, 1 or 2 times a week, 1 to 3 times a month, special occasions only, never, or unknown), smoking status (never, former, current, or unknown), physical activity (low, moderate, high, or unknown), total energy intake (continuous), polygenic risk score for CRC (continuous), first 10 principal components of ancestry (in Units, continuous), and genotype measurement batch (continuous).

Abbreviations: CI, confidence interval; CRC, colorectal cancer; hPDI, healthful plant-based diet index; HR, hazard ratio; PDI, plant-based diet index; uPDI, unhealthful plant-based diet index.

**
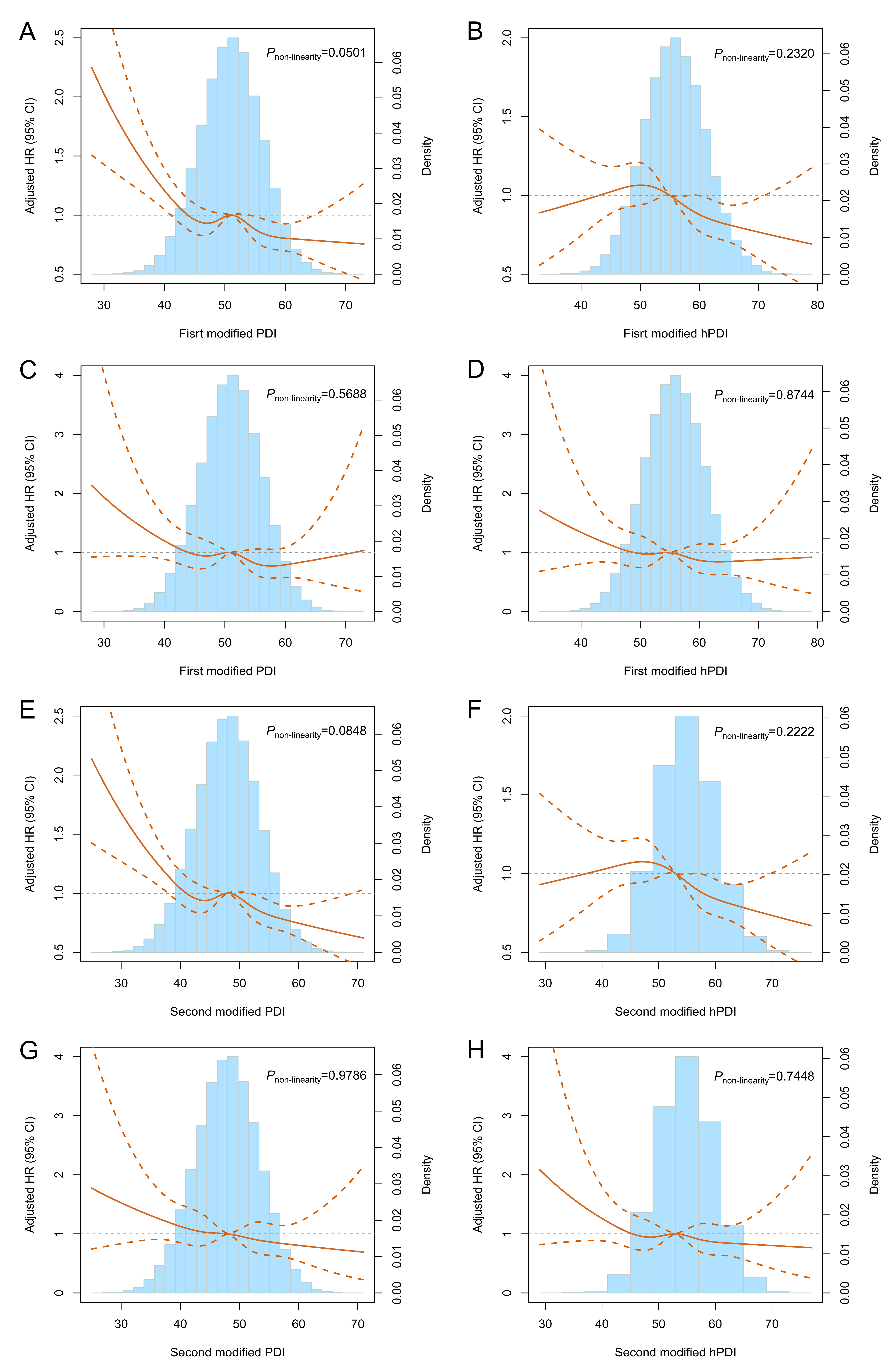
**

**Figure S5.** Restricted cubic splines for the modified PDI/hPDI and risks of CRC incidence and mortality

(A) The first modified PDI and CRC incidence; (B) The first modified hPDI and CRC incidence; (C) The first modified PDI and CRC mortality; (D) The first modified hPDI and CRC mortality; (E) The second modified PDI and CRC incidence; (F) The second modified hPDI and CRC incidence; (G) The second modified PDI and CRC mortality; (H) The second modified hPDI and CRC mortality.

The models adjusted for age (continuous), sex (female, male), ethnicity (White, mixed, Asian, Black, Chinese, others, or unknown), education (college or university, vocational qualification, upper secondary, lower secondary, others, or unknown), Townsend deprivation index (in quintiles), body mass index (<18.5, 18.5-24.9, 25-29.9, or ≥30 kg/m2), alcohol frequency (daily or almost daily, 3 or 4 times a week, 1 or 2 times a week, 1 to 3 times a month, special occasions only, never, or unknown), smoking status (never, former, current, or unknown), physical activity (low, moderate, high, or unknown), total energy intake (continuous), polygenic risk score for CRC (continuous), first 10 principal components of ancestry (in Units, continuous), and genotype measurement batch (continuous).

Abbreviations: CI, confidence interval; CRC, colorectal cancer; hPDI, healthful plant-based diet index; HR, hazard ratio; PDI, plant-based diet index.

**Table S1. Definition of CRC in the UK Biobank Study**

|  | **ICD-9**  (Field ID: 40013, 41271) | **ICD-10**  (Field ID: 40001, 40002, 41270, 40006) | **Self-reported**  (Field ID: 20001) |
| --- | --- | --- | --- |
| **CRC** | 153-154 | C18-C20 | 1020, 1022, 1023, 1086 |
| **Proximal colon cancer**  **(cecum, appendix, ascending colon, hepatic flexure, transverse colon, splenic flexure)** | 1530, 1531, 1534-1537 | C18.0-C18.5 | 1086 |
| **Distal colon cancer**  **(descending and sigmoid colons)** | 1532, 1533 | C18.6-C18.7 | / |
| **Rectal cancer**  **(rectosigmoid junction, rectum)** | 1540, 1541 | C19-C20 | 1023 |

Abbreviations: CRC, colorectal cancer; ICD, International Classification of Diseases.

**Table S2. Examples of food items constituting the 17 food groups in UK Biobank study**

| **Food groups** | **Food items** | **PDI scoring** | **hPDI scoring** | **uPDI scoring** |
| --- | --- | --- | --- | --- |
| ***Healthy Plant Food Groups*** | | | | |
| Whole grains | Porridge, hot oat cereal, muesli, plain cereals, bran cereals, whole-wheat cereal, sliced bread, bap, bread roll, oatcakes, wholemeal pasta, brown rice, other cooked grains (e.g. bulgur wheat, millet or pearl barley) | Positive | Positive | Reverse |
| Fruits | Stewed fruit, prune, dried fruit, mixed fruit, apple, banana, berry, cherry, grapefruit, grape, mango, melon, orange, satsuma, peach/nectarine, pear, pineapple, plum, other fruit (e.g. pomegranate, kiwi, papaya) | Positive | Positive | Reverse |
| Vegetables | Mixed vegetable, vegetable pieces, coleslaw, side salad, avocado, beetroot, broccoli, butternut squash, cabbage/kale, carrot, cauliflower, celery, courgetti, cucumber, garlic, leek, lettuce, mushroom, onion, parsnip, sweet pepper, spinach, sprouts, sweetcorn, sweet potato, fresh tomato, tinned tomato, turnip/swede, watercress, other vegetables (e.g. celeriac, asparagus, fennel, aubergine, pumpkin) | Positive | Positive | Reverse |
| Nuts | Salted peanuts, unsalted peanuts, salted nuts, unsalted nuts, seeds | Positive | Positive | Reverse |
| Legumes | Vegetarian sausages/burgers, tofu, quorn, other vegetarian alternative (e.g. nut roast), baked beans, pulses, broad beans, green beans, peas | Positive | Positive | Reverse |
| Tea and coffee | Instant coffee, filtered coffee, cappuccino, latte, espresso, other coffee types, standard tea, rooibos tea, green tea, herbal tea, other tea or infusion | Positive | Positive | Reverse |
| ***Less Healthy Plant Food Groups*** | | | | |
| Refined grains | Oat crunch, sweetened cereal, other cereal, naan bread, garlic bread, crispbread, other bread (e.g. crumpets, tortilla wraps, breadsticks), white pasta, white rice, snackpot, couscous, pancake, scotch pancake, croissant, scone, savoury biscuits, cheesy biscuits, other savoury snacks | Positive | Reverse | Positive |
| Potatoes | Crisps, fried potatoes, boiled/baked potatoes, mashed potatoes | Positive | Reverse | Positive |
| Sugary drinks | Low calorie or diet drinks, fizzy drinks, squash or cordial | Positive | Reverse | Positive |
| Fruit juices | Orange juice, grapefruit juice, pure fruit/vegetable juice, fruit smoothie | Positive | Reverse | Positive |
| Sweets and desserts | Double crust pastry, single crust pastry, crumble, Yorkshire pudding, Danish pastry, soya dessert, fruitcake, cake, doughnut, sponge pudding, other dessert, chocolate bar, white chocolate, milk chocolate, dark chocolate, chocolate-covered raisin, chocolate sweets, low sugar/sugar free sweets, sweets (e.g. peppermints, toffees, fudge, fruit flavoured sweets), chocolate-covered biscuits, chocolate biscuits (e.g. chocolate digestive biscuits), sweet biscuits (e.g. digestive, shortbread, ginger nut), cereal bar, other sweets | Positive | Reverse | Positive |
| ***Animal Food Groups*** | | | | |
| Animal fat | Butter/margarine on bread slices, baguettes, baps, bread rolls, crackers/crispbreads, oatcakes, other bread types | Reverse | Reverse | Reverse |
| Dairy | Low fat hard cheese, hard cheese, soft cheese, blue cheese, low fat cheese spread, cheese spread, cottage cheese, feta, mozzarella, goat's cheese, other cheese | Reverse | Reverse | Reverse |
| Eggs | Whole egg, omelette, eggs in sandwiches, scotch egg, other egg | Reverse | Reverse | Reverse |
| Fish or seafood | Tinned tuna, oily fish, breaded fish, battered fish, white fish, prawns, lobster/crab, shellfish, other fish | Reverse | Reverse | Reverse |
| Meat | Sausage, beef, pork, lamb, crumbed or deep-fried poultry, poultry, bacon, ham, liver, other meat (e.g. duck, goose, kidney) | Reverse | Reverse | Reverse |
| Miscellaneous animal-based foods | Pizza, Indian snacks | Reverse | Reverse | Reverse |

Abbreviations: PDI, overall plant-based diet index; hPDI, healthful plant-based diet index; uPDI, unhealthful plant-based diet index.

**Table S3. Scores of food items of 186675 participants by plant-based diet indices groups**

|  | **Plant-based Diet Indices Groups** | | | |
| --- | --- | --- | --- | --- |
|  | **Q1** | **Q2** | **Q3** | **Q4** |
| ***Overall plant-based diet index (PDI)*** | | | | |
| Healthy plant food groups | 11.94 (2.85) | 14.32 (2.94) | 15.96 (2.99) | 18.36 (3.22) |
| Whole grains | 2.40 (1.35) | 2.90 (1.36) | 3.19 (1.33) | 3.49 (1.27) |
| Fruits | 2.21 (1.25) | 2.79 (1.36) | 3.20 (1.35) | 3.74 (1.26) |
| Vegetables | 2.21 (1.28) | 2.82 (1.35) | 3.21 (1.33) | 3.73 (1.25) |
| Nuts | 1.19 (0.56) | 1.29 (0.67) | 1.39 (0.77) | 1.60 (0.98) |
| Legumes | 1.39 (0.77) | 1.60 (0.93) | 1.79 (1.07) | 2.28 (1.36) |
| Tea and coffee | 2.54 (1.37) | 2.92 (1.42) | 3.18 (1.42) | 3.52 (1.40) |
| Less healthy plant food groups | 10.70 (2.72) | 12.37 (2.75) | 13.36 (2.82) | 14.75 (2.98) |
| Refined grains | 2.50 (1.51) | 2.92 (1.50) | 3.14 (1.46) | 3.45 (1.40) |
| Potatoes | 2.53 (1.41) | 2.93 (1.38) | 3.11 (1.35) | 3.32 (1.33) |
| Sugary drinks | 1.70 (1.10) | 1.81 (1.15) | 1.90 (1.21) | 2.07 (1.30) |
| Fruit juices | 1.65 (1.15) | 2.00 (1.34) | 2.26 (1.44) | 2.67 (1.54) |
| Sweets and desserts | 2.54 (1.37) | 2.95 (1.38) | 3.16 (1.35) | 3.41 (1.33) |
| Animal food groups | 15.38 (2.77) | 14.10 (2.68) | 13.35 (2.66) | 12.25 (2.72) |
| Animal fat | 3.44 (1.38) | 3.13 (1.40) | 2.94 (1.41) | 2.63 (1.42) |
| Dairy | 3.76 (1.46) | 3.64 (1.49) | 3.53 (1.52) | 3.36 (1.58) |
| Eggs | 1.91 (1.23) | 1.61 (0.94) | 1.50 (0.84) | 1.40 (0.74) |
| Fish or seafood | 1.75 (1.02) | 1.69 (0.90) | 1.67 (0.86) | 1.62 (0.83) |
| Meat | 3.39 (1.43) | 3.12 (1.37) | 2.92 (1.35) | 2.59 (1.34) |
| Miscellaneous animal-based foods | 1.26 (0.82) | 1.13 (0.56) | 1.09 (0.45) | 1.06 (0.37) |
| ***Healthful plant-based diet index (hPDI)*** | | | | |
| Healthy plant food groups | 12.22 (2.95) | 14.26 (3.07) | 15.83 (3.10) | 18.39 (3.25) |
| Whole grains | 2.38 (1.32) | 2.87 (1.36) | 3.18 (1.33) | 3.56 (1.25) |
| Fruits | 2.27 (1.26) | 2.76 (1.36) | 3.17 (1.37) | 3.78 (1.27) |
| Vegetables | 2.41 (1.29) | 2.80 (1.36) | 3.13 (1.38) | 3.66 (1.33) |
| Nuts | 1.20 (0.55) | 1.29 (0.67) | 1.38 (0.76) | 1.61 (0.99) |
| Legumes | 1.46 (0.81) | 1.61 (0.95) | 1.78 (1.09) | 2.24 (1.37) |
| Tea and coffee | 2.50 (1.34) | 2.93 (1.41) | 3.19 (1.43) | 3.56 (1.40) |
| Less healthy plant food groups | 15.48 (2.77) | 13.44 (2.60) | 12.14 (2.54) | 10.45 (2.53) |
| Refined grains | 3.49 (1.43) | 3.13 (1.49) | 2.88 (1.49) | 2.68 (1.48) |
| Potatoes | 3.48 (1.34) | 3.12 (1.36) | 2.87 (1.36) | 2.49 (1.33) |
| Sugary drinks | 2.47 (1.41) | 1.93 (1.19) | 1.68 (1.05) | 1.43 (0.85) |
| Fruit juices | 2.59 (1.54) | 2.26 (1.45) | 2.04 (1.37) | 1.76 (1.23) |
| Sweets and desserts | 3.61 (1.29) | 3.21 (1.34) | 2.90 (1.35) | 2.42 (1.31) |
| Animal food groups | 15.72 (2.61) | 14.26 (2.55) | 13.27 (2.54) | 11.80 (2.59) |
| Animal fat | 3.62 (1.30) | 3.22 (1.37) | 2.89 (1.39) | 2.39 (1.38) |
| Dairy | 3.92 (1.37) | 3.66 (1.47) | 3.48 (1.54) | 3.21 (1.60) |
| Eggs | 1.81 (1.13) | 1.62 (0.97) | 1.54 (0.90) | 1.44 (0.81) |
| Fish or seafood | 1.71 (0.96) | 1.68 (0.89) | 1.67 (0.88) | 1.65 (0.87) |
| Meat | 3.56 (1.34) | 3.16 (1.37) | 2.87 (1.35) | 2.43 (1.31) |
| Miscellaneous animal-based foods | 1.23 (0.77) | 1.14 (0.59) | 1.10 (0.50) | 1.06 (0.39) |
| ***Unhealthful plant-based diet index (uPDI)*** | | | | |
| Healthy plant food groups | 18.93 (3.04) | 16.31 (2.83) | 14.40 (2.80) | 11.86 (2.76) |
| Whole grains | 3.75 (1.27) | 3.26 (1.32) | 2.86 (1.31) | 2.29 (1.22) |
| Fruits | 3.82 (1.24) | 3.26 (1.34) | 2.83 (1.36) | 2.23 (1.26) |
| Vegetables | 3.79 (1.28) | 3.24 (1.35) | 2.84 (1.35) | 2.30 (1.26) |
| Nuts | 1.61 (1.00) | 1.40 (0.78) | 1.31 (0.69) | 1.21 (0.57) |
| Legumes | 2.21 (1.34) | 1.85 (1.13) | 1.65 (1.00) | 1.47 (0.86) |
| Tea and coffee | 3.75 (1.32) | 3.30 (1.40) | 2.91 (1.40) | 2.36 (1.30) |
| Less healthy plant food groups | 10.71 (2.70) | 12.22 (2.74) | 13.21 (2.83) | 14.86 (2.98) |
| Refined grains | 2.56 (1.49) | 2.92 (1.50) | 3.09 (1.49) | 3.40 (1.43) |
| Potatoes | 2.64 (1.36) | 2.89 (1.36) | 3.05 (1.38) | 3.29 (1.39) |
| Sugary drinks | 1.48 (0.88) | 1.68 (1.05) | 1.89 (1.19) | 2.37 (1.40) |
| Fruit juices | 1.78 (1.23) | 2.05 (1.37) | 2.24 (1.44) | 2.49 (1.54) |
| Sweets and desserts | 2.51 (1.34) | 2.91 (1.37) | 3.13 (1.36) | 3.46 (1.34) |
| Animal food groups | 15.56 (2.68) | 14.34 (2.58) | 13.38 (2.62) | 11.96 (2.66) |
| Animal fat | 3.40 (1.41) | 3.17 (1.40) | 2.97 (1.41) | 2.62 (1.40) |
| Dairy | 4.16 (1.24) | 3.81 (1.40) | 3.45 (1.52) | 2.90 (1.59) |
| Eggs | 1.91 (1.19) | 1.65 (0.97) | 1.52 (0.88) | 1.36 (0.74) |
| Fish or seafood | 1.86 (1.02) | 1.71 (0.90) | 1.64 (0.86) | 1.54 (0.81) |
| Meat | 3.19 (1.45) | 3.06 (1.41) | 2.96 (1.38) | 2.82 (1.36) |
| Miscellaneous animal-based foods | 1.16 (0.63) | 1.14 (0.59) | 1.13 (0.57) | 1.11 (0.53) |

Data for 3 food categories (healthy plant food groups, less Healthy plant food groups, and animal food groups) and 17 food groups were expressed as mean (standard deviation) of scores.

**Table S4. List of 95 SNPs included in the polygenic risk score for CRC**

| **SNP** | **Locus** | **Chr** | **Position (hg19)** | **Risk allele** | **RAF** | **Weight (beta)** | ***P* value** |
| --- | --- | --- | --- | --- | --- | --- | --- |
| rs12144319 | 1p32.3 | 1 | 55246035 | C | 0.25 | 0.068 | 3.30E-11 |
| rs4360494 | 1p34.3 | 1 | 38455891 | G | 0.45 | 0.049 | 3.80E-09 |
| rs10911251 | 1q25.3 | 1 | 183081194 | A | 0.55 | 0.068 | 4.93E-08 |
| rs6687758 | 1q41 | 1 | 222164948 | G | 0.22 | 0.083 | 1.47E-08 |
| rs448513 | 2q24.2 | 2 | 159964552 | C | 0.33 | 0.049 | 4.40E-08 |
| rs11903757 | 2q32.3 | 2 | 192587204 | C | 0.17 | 0.051 | 4.86E-03 |
| rs983402 | 2q33.1 | 2 | 199781586 | T | 0.33 | 0.068 | 7.70E-12 |
| rs11884596 | 2q33.1 | 2 | 199612407 | C | 0.38 | 0.058 | 1.10E-09 |
| rs992157 | 2q35 | 2 | 219154781 | A | 0.58 | 0.068 | 1.41E-07 |
| rs812481 | 3p14.1 | 3 | 66442435 | G | 0.56 | 0.062 | 1.11E-06 |
| rs35360328 | 3p22.1 | 3 | 40924962 | A | 0.16 | 0.122 | 6.21E-13 |
| rs72942485 | 3q13.2 | 3 | 112999560 | G | 0.98 | 0.174 | 2.10E-08 |
| rs10049390 | 3q22.2 | 3 | 133701119 | A | 0.74 | 0.058 | 3.80E-09 |
| rs10936599 | 3q26.2 | 3 | 169492101 | C | 0.76 | 0.03 | 2.57E-02 |
| rs1370821 | 4q22.2 | 4 | 94943383 | T | 0.40 | 0.068 | 4.00E-08 |
| rs1391441 | 4q24 | 4 | 106128760 | A | 0.67 | 0.049 | 1.60E-08 |
| rs11727676 | 4q31.21 | 4 | 145659064 | C | 0.10 | 0.086 | 2.90E-08 |
| rs58791712 | 5p13.1 | 5 | 40281798 | GT | 0.26 | 0.094 | 7.30E-14 |
| rs2735940 | 5p15.33 | 5 | 1296486 | G | 0.49 | 0.083 | 3.10E-13 |
| rs78368589 | 5p15.33 | 5 | 1240204 | T | 0.06 | 0.131 | 9.40E-12 |
| rs145364999 | 5q21.1 | 5 | 98206082 | T | 1.00 | 0.554 | 6.30E-09 |
| rs647161 | 5q31.1 | 5 | 134499092 | A | 0.66 | 0.058 | 1.21E-05 |
| rs62404968 | 6p12.1 | 6 | 55714314 | C | 0.75 | 0.083 | 8.60E-10 |
| rs1321311 | 6p21.2 | 6 | 36622900 | A | 0.22 | 0.049 | 3.63E-04 |
| rs6906359 | 6p21.31 | 6 | 35528378 | C | 0.90 | 0.105 | 3.40E-08 |
| rs9271695 | 6p21.32 | 6 | 32593080 | G | 0.80 | 0.086 | 1.10E-13 |
| rs12672022 | 7p13 | 7 | 45136423 | T | 0.83 | 0.068 | 2.80E-08 |
| rs140355816 | 8q23.3 | 8 | 117574515 | G | 0.01 | 0.198 | 4.97E-06 |
| rs2450115 | 8q23.3 | 8 | 117624093 | T | 0.81 | 0.077 | 3.40E-07 |
| rs16892766 | 8q23.3 | 8 | 117630683 | C | 0.09 | 0.198 | 3.94E-24 |
| rs6983267 | 8q24.21 | 8 | 128413305 | G | 0.50 | 0.128 | 7.74E-27 |
| rs4313119 | 8q24.21 | 8 | 128571855 | G | 0.75 | 0.058 | 1.00E-09 |
| rs1537372 | 9p21.3 | 9 | 22103183 | G | 0.57 | 0.049 | 1.40E-08 |
| rs719725 | 9p24.1 | 9 | 6365683 | A | 0.59 | 0.039 | 4.50E-04 |
| rs34405347 | 9q22.33 | 9 | 101679752 | T | 0.90 | 0.086 | 3.10E-08 |
| rs10980628 | 9q31.3 | 9 | 113671403 | C | 0.21 | 0.068 | 2.80E-09 |
| rs11255841 | 10p14 | 10 | 8739580 | T | 0.69 | 0.094 | 3.16E-12 |
| rs10994860 | 10q11.23 | 10 | 52645424 | C | 0.80 | 0.083 | 3.50E-08 |
| rs704017 | 10q22.3 | 10 | 80819132 | G | 0.56 | 0.073 | 1.96E-08 |
| rs1035209 | 10q24.2 | 10 | 101345366 | T | 0.20 | 0.086 | 1.03E-08 |
| rs4919687 | 10q24.32 | 10 | 104595248 | G | 0.70 | 0.03 | 7.79E-03 |
| rs12241008 | 10q25.2 | 10 | 114280702 | C | 0.10 | 0.062 | 1.60E-03 |
| rs11196172 | 10q25.2 | 10 | 114726843 | A | 0.12 | 0.058 | 6.05E-04 |
| rs1535 | 11q12.2 | 11 | 61597972 | A | 0.65 | 0.068 | 4.15E-08 |
| rs3824999 | 11q13.4 | 11 | 74345550 | G | 0.52 | 0.073 | 3.81E-10 |
| rs61389091 | 11q13.4 | 11 | 74427921 | C | 0.96 | 0.207 | 1.20E-18 |
| rs2186607 | 11q22.1 | 11 | 101656397 | T | 0.52 | 0.049 | 1.50E-09 |
| rs3802842 | 11q23.1 | 11 | 111171709 | C | 0.27 | 0.117 | 5.30E-19 |
| rs10849432 | 12p13.31 | 12 | 6385727 | T | 0.90 | 0.086 | 2.34E-05 |
| rs10774214 | 12p13.32 | 12 | 4368352 | T | 0.38 | 0.039 | 4.29E-04 |
| rs3217810 | 12p13.32 | 12 | 4388271 | T | 0.12 | 0.14 | 1.61E-11 |
| rs3217874 | 12p13.32 | 12 | 4400808 | T | 0.43 | 0.077 | 1.20E-17 |
| rs11610543 | 12q12 | 12 | 43134191 | G | 0.50 | 0.049 | 1.30E-09 |
| rs34245511 | 12q13.12 | 12 | 50573433 | C | 0.36 | 0.049 | 3.00E-04 |
| rs11169552 | 12q13.12 | 12 | 51155663 | C | 0.75 | 0.051 | 3.61E-04 |
| rs4759277 | 12q13.3 | 12 | 57533690 | A | 0.35 | 0.049 | 9.40E-09 |
| rs3184504 | 12q24.12 | 12 | 111884608 | C | 0.54 | 0.073 | 1.94E-10 |
| rs59336 | 12q24.21 | 12 | 115116352 | T | 0.49 | 0.051 | 1.34E-05 |
| rs12822984 | 12q24.21 | 12 | 115888504 | A | 0.50 | 0.073 | 5.00E-11 |
| rs73208120 | 12q24.22 | 12 | 117747590 | G | 0.08 | 0.105 | 7.59E-07 |
| rs10161980 | 13q13.2 | 13 | 34093518 | C | 0.62 | 0.077 | 4.70E-09 |
| rs7333607 | 13q13.3 | 13 | 37462010 | G | 0.24 | 0.077 | 6.30E-13 |
| rs78341008 | 13q22.1 | 13 | 73791554 | C | 0.07 | 0.113 | 3.20E-10 |
| rs8000189 | 13q34 | 13 | 111075881 | T | 0.64 | 0.058 | 1.80E-09 |
| rs4444235 | 14q22.2 | 14 | 54410919 | C | 0.49 | 0.073 | 1.09E-09 |
| rs1957636 | 14q22.2 | 14 | 54560018 | T | 0.41 | 0.049 | 6.42E-05 |
| rs17094983 | 14q23.1 | 14 | 59189361 | G | 0.88 | 0.086 | 4.60E-11 |
| rs16969681 | 15q13.3 | 15 | 32993111 | T | 0.07 | 0.095 | 1.27E-06 |
| rs11632715 | 15q13.3 | 15 | 33004247 | A | 0.46 | 0.058 | 7.68E-07 |
| rs73376930 | 15q13.3 | 15 | 33012502 | G | 0.21 | 0.128 | 3.57E-18 |
| rs17816465 | 15q13.3 | 15 | 33156386 | A | 0.21 | 0.068 | 6.80E-09 |
| rs56324967 | 15q22.33 | 15 | 67402824 | C | 0.68 | 0.068 | 1.10E-13 |
| rs9929218 | 16q22.1 | 16 | 68820946 | G | 0.71 | 0.062 | 7.30E-06 |
| rs9930005 | 16q23.2 | 16 | 80043258 | C | 0.43 | 0.049 | 2.10E-08 |
| rs2696839 | 16q24.1 | 16 | 86340448 | G | 0.51 | 0.062 | 2.00E-08 |
| rs1078643 | 17p12 | 17 | 10707241 | A | 0.76 | 0.077 | 6.60E-12 |
| rs12603526 | 17p13.3 | 17 | 800593 | C | 0.01 | 0.105 | 2.47E-02 |
| rs983318 | 17q24.3 | 17 | 70413253 | A | 0.25 | 0.058 | 5.60E-09 |
| rs75954926 | 17q25.3 | 17 | 81061048 | G | 0.66 | 0.086 | 3.00E-18 |
| rs7229639 | 18q21.1 | 18 | 46450976 | A | 0.10 | 0.077 | 6.92E-05 |
| rs4939827 | 18q21.1 | 18 | 46453463 | T | 0.53 | 0.131 | 3.41E-30 |
| rs34797592 | 19p13.11 | 19 | 16417198 | T | 0.12 | 0.086 | 4.20E-10 |
| rs10411210 | 19q13.11 | 19 | 33532300 | C | 0.90 | 0.094 | 3.44E-07 |
| rs1800469 | 19q13.2 | 19 | 41860296 | G | 0.69 | 0.03 | 9.59E-03 |
| rs73068325 | 19q13.43 | 19 | 59079096 | T | 0.18 | 0.068 | 4.20E-08 |
| rs961253 | 20p12.3 | 20 | 6404281 | A | 0.36 | 0.077 | 5.79E-11 |
| rs4813802 | 20p12.3 | 20 | 6699595 | G | 0.32 | 0.073 | 1.24E-09 |
| rs2423279 | 20p12.3 | 20 | 7812350 | C | 0.27 | 0.062 | 1.57E-05 |
| rs28488 | 20p12.3 | 20 | 6762221 | T | 0.64 | 0.058 | 2.60E-11 |
| rs994308 | 20p12.3 | 20 | 6603622 | C | 0.59 | 0.077 | 4.80E-18 |
| rs2295444 | 20q11.22 | 20 | 33173883 | C | 0.51 | 0.073 | 3.30E-09 |
| rs6031311 | 20q13.12 | 20 | 42666475 | T | 0.76 | 0.058 | 6.80E-09 |
| rs6066825 | 20q13.13 | 20 | 47340117 | A | 0.62 | 0.058 | 2.29E-06 |
| rs1810502 | 20q13.13 | 20 | 49057488 | C | 0.55 | 0.073 | 1.02E-08 |
| rs2427308 | 20q13.33 | 20 | 60969451 | C | 0.77 | 0.117 | 1.75E-13 |

Abbreviations: Chr, Chromosome; CRC, colorectal cancer; RAF, risk allele frequency; SNP, single nucleotide polymorphism.

**Table S5. Baseline characteristics of 186675 participants by hPDI groups**

|  | **Healthful Plant-based Diet Index (hPDI)** | | | |
| --- | --- | --- | --- | --- |
|  | **Q1** | **Q2** | **Q3** | **Q4** |
| Range, scores | 29-50 | 51-54 | 55-58 | 59-82 |
| Number of participants | 45253 | 46602 | 47217 | 47603 |
| Age, mean (SD), years | 56.8 (8.3) | 58.2 (8.1) | 58.7 (7.9) | 58.8 (7.7) |
| Male, n (%) | 26031 (57.5) | 22576 (48.4) | 20121 (42.6) | 16694 (35.1) |
| Ethnicity, n (%) |  |  |  |  |
| White | 43199 (95.5) | 44550 (95.6) | 45134 (95.6) | 45076 (94.7) |
| Mixed | 303 (0.7) | 271 (0.6) | 256 (0.5) | 296 (0.6) |
| Asian | 508 (1.1) | 595 (1.3) | 679 (1.4) | 924 (1.9) |
| Black | 702 (1.6) | 561 (1.2) | 519 (1.1) | 527 (1.1) |
| Chinese | 100 (0.2) | 114 (0.2) | 131 (0.3) | 198 (0.4) |
| Others | 291 (0.6) | 332 (0.7) | 335 (0.7) | 428 (0.9) |
| Unknown | 150 (0.3) | 179 (0.4) | 163 (0.4) | 154 (0.3) |
| Education, n (%) |  |  |  |  |
| College or university | 17586 (38.9) | 19280 (41.4) | 20398 (43.2) | 22484 (47.2) |
| Vocational | 4799 (10.6) | 4876 (10.5) | 4848 (10.3) | 4852 (10.2) |
| Upper secondary | 6236 (13.8) | 6065 (13.0) | 6124 (13.0) | 6035 (12.7) |
| Lower secondary | 12587 (27.8) | 12037 (25.8) | 11629 (24.6) | 10396 (21.8) |
| Others | 3829 (8.5) | 4109 (8.8) | 3989 (8.5) | 3608 (7.6) |
| Unknown | 216 (0.5) | 235 (0.5) | 229 (0.5) | 228 (0.5) |
| Townsend deprivation index, median (IQR) | −2.3 (−3.7 to 0.2) | −2.4 (−3.7 to 0) | −2.4 (−3.8 to 0) | −2.3 (−3.7 to 0.1) |
| Body mass index, n (%) |  |  |  |  |
| <18.5 | 151 (0.3) | 204 (0.4) | 282 (0.6) | 364 (0.8) |
| 18.5~24.9 | 13374 (29.6) | 16439 (35.3) | 18214 (38.6) | 20586 (43.3) |
| 25~29.9 | 19585 (43.3) | 19772 (42.4) | 19480 (41.3) | 18654 (39.2) |
| ≥30 | 12022 (26.6) | 10052 (21.6) | 9130 (19.3) | 7854 (16.5) |
| Unknown | 121 (0.3) | 135 (0.3) | 111 (0.2) | 145 (0.3) |
| Alcohol consumption, n (%) |  |  |  |  |
| Daily or almost daily | 10355 (22.9) | 10926 (23.5) | 11002 (23.3) | 10114 (21.3) |
| 3 or 4 times a week | 11007 (24.3) | 11847 (25.4) | 12141 (25.7) | 11987 (25.2) |
| 1 or 2 times a week | 11434 (25.3) | 11652 (25.0) | 11702 (24.8) | 11867 (24.9) |
| 1 to 3 times a month | 5277 (11.7) | 5011 (10.8) | 5080 (10.8) | 5244 (11.0) |
| Special occasions only | 4409 (9.7) | 4432 (9.5) | 4428 (9.4) | 4979 (10.5) |
| Never | 2731 (6.0) | 2697 (5.8) | 2820 (6.0) | 3374 (7.1) |
| Unknown | 40 (0.1) | 37 (0.1) | 44 (0.1) | 38 (0.1) |
| Smoking status, n (%) |  |  |  |  |
| Never | 25525 (56.4) | 26668 (57.2) | 26782 (56.7) | 27286 (57.3) |
| Former smokers | 15412 (34.1) | 16049 (34.4) | 16763 (35.5) | 17127 (36.0) |
| Current smokers | 4209 (9.3) | 3751 (8.1) | 3547 (7.5) | 3070 (6.5) |
| Unknown | 107 (0.2) | 134 (0.3) | 125 (0.3) | 120 (0.3) |
| Physical activity, n (%) |  |  |  |  |
| Low | 8502 (18.8) | 7743 (16.6) | 6928 (14.7) | 5948 (12.5) |
| Moderate | 16307 (36.0) | 16828 (36.1) | 17061 (36.1) | 16792 (35.3) |
| High | 13631 (30.1) | 14792 (31.7) | 16029 (34.0) | 17869 (37.5) |
| Unknown | 6813 (15.1) | 7239 (15.5) | 7199 (15.3) | 6994 (14.7) |
| Energy intake, mean (SD), kcal/d | 2306.3 (548.7) | 2081.2 (515.7) | 1962.6 (508.4) | 1836.8 (500.7) |

Data were expressed as mean (SD) or number of participants (proportion). Nonparametric tests were used for continuous variables and chi-square tests were used for categorical variables. All tests had *P* values less than 0.001.

Abbreviations: CRC, colorectal cancer; IQR, inter-quartile range; SD, standard deviation.

**Table S6. Baseline characteristics of 186675 participants by uPDI groups**

|  | **Unhealthful Plant-based Diet Index (uPDI)** | | | |
| --- | --- | --- | --- | --- |
|  | **Q1** | **Q2** | **Q3** | **Q4** |
| Range, scores | 28-51 | 52-55 | 56-59 | 60-79 |
| Number of participants | 41776 | 46868 | 48592 | 49439 |
| Age, mean (SD), years | 59.8 (7.4) | 59.0 (7.8) | 58.1 (8.0) | 55.9 (8.2) |
| Male, n (%) | 18292 (43.8) | 20944 (44.7) | 22399 (46.1) | 23787 (48.1) |
| Ethnicity, n (%) |  |  |  |  |
| White | 40452 (96.8) | 45148 (96.3) | 46294 (95.3) | 46065 (93.2) |
| Mixed | 208 (0.5) | 255 (0.5) | 266 (0.6) | 397 (0.8) |
| Asian | 383 (0.9) | 516 (1.1) | 726 (1.5) | 1081 (2.2) |
| Black | 225 (0.5) | 381 (0.8) | 588 (1.2) | 1115 (2.3) |
| Chinese | 116 (0.3) | 117 (0.3) | 143 (0.3) | 167 (0.3) |
| Others | 242 (0.6) | 300 (0.6) | 392 (0.8) | 452 (0.9) |
| Unknown | 150 (0.4) | 151 (0.3) | 183 (0.4) | 162 (0.3) |
| Education, n (%) |  |  |  |  |
| College or university | 19232 (46.0) | 20738 (44.3) | 20410 (42.0) | 19368 (39.2) |
| Vocational | 4339 (10.4) | 4885 (10.4) | 5097 (10.5) | 5054 (10.2) |
| Upper secondary | 5345 (12.8) | 6036 (12.9) | 6370 (13.1) | 6709 (13.8) |
| Lower secondary | 9360 (22.4) | 11112 (23.7) | 12245 (25.2) | 13932 (28.2) |
| Others | 3332 (8.0) | 3881 (8.3) | 4225 (8.7) | 4097 (8.3) |
| Unknown | 168 (0.4) | 216 (0.5) | 245 (0.5) | 279 (0.6) |
| Townsend deprivation index, median (IQR) | −2.4 (−3.8 to −0.1) | −2.4 (−3.8 to −0.1) | −2.3 (−3.7 to 0.1) | −2.2 (−3.7 to 0.4) |
| Body mass index, n (%) |  |  |  |  |
| <18.5 | 251 (0.6) | 288 (0.6) | 231 (0.5) | 231 (0.5) |
| 18.5~24.9 | 16251 (38.9) | 17800 (38.0) | 17853 (36.7) | 16709 (33.8) |
| 25~29.9 | 17062 (40.8) | 19405 (41.4) | 20241 (41.7) | 20783 (42.0) |
| ≥30 | 8108 (19.4) | 9254 (19.7) | 10144 (20.9) | 11552 (23.4) |
| Unknown | 104 (0.3) | 121 (0.3) | 123 (0.3) | 164 (0.3) |
| Alcohol consumption, n (%) |  |  |  |  |
| Daily or almost daily | 9244 (22.1) | 10731 (22.9) | 11450 (23.6) | 10972 (22.2) |
| 3 or 4 times a week | 10832 (25.9) | 12242 (26.1) | 12347 (25.4) | 11561 (23.4) |
| 1 or 2 times a week | 10489 (25.1) | 11702 (25.0) | 12046 (24.8) | 12418 (25.1) |
| 1 to 3 times a month | 4640 (11.1) | 5068 (10.8) | 5209 (10.7) | 5695 (11.5) |
| Special occasions only | 4108 (9.8) | 4394 (9.4) | 4575 (9.4) | 5171 (10.5) |
| Never | 2440 (5.8) | 2698 (5.8) | 2916 (6.0) | 3568 (7.2) |
| Unknown | 23 (0.1) | 33 (0.1) | 49 (0.1) | 54 (0.1) |
| Smoking status, n (%) |  |  |  |  |
| Never | 23299 (55.8) | 26458 (56.5) | 27733 (57.1) | 28771 (58.2) |
| Former smokers | 15667 (37.5) | 16895 (36.1) | 16933 (34.9) | 15856 (32.1) |
| Current smokers | 2721 (6.5) | 3404 (7.3) | 3800 (7.8) | 4652 (9.4) |
| Unknown | 89 (0.2) | 111 (0.2) | 126 (0.3) | 160 (0.3) |
| Physical activity, n (%) |  |  |  |  |
| Low | 5349 (12.8) | 6742 (14.4) | 7766 (16.0) | 9264 (18.7) |
| Moderate | 14722 (35.2) | 16996 (36.3) | 17703 (36.4) | 17567 (35.5) |
| High | 15640 (37.4) | 16170 (34.5) | 15694 (32.3) | 14817 (30.0) |
| Unknown | 6065 (14.5) | 6960 (14.9) | 7429 (15.3) | 7791 (15.8) |
| Energy intake, mean (SD), kcal/d | 2145.0 (547.4) | 2067.2 (534.8) | 2014.0 (536.0) | 1964.2 (550.8) |

Data were expressed as mean (SD) or number of participants (proportion). Nonparametric tests were used for continuous variables and chi-square tests were used for categorical variables. All tests had *P* values less than 0.001.

Abbreviations: CRC, colorectal cancer; IQR, inter-quartile range; SD, standard deviation.

**Table S7. Association between plant-based diet indices and risk of CRC incidence**

| **Plant-based diet indices** | **Cases/person-years** | **Incident rate per 1000 person-years** | **HR (95% CI)** | | |
| --- | --- | --- | --- | --- | --- |
|  |  |  | **Model 1** | **Model 2** | **Model 3** |
| ***PDI*** |  |  |  |  |  |
| Q1 (24-46) | 555/433782 | 1.28 | 1.00 (ref.) | 1.00 (ref.) | 1.00 (ref.) |
| Q2 (47-49) | 605/499171 | 1.21 | 0.92 (0.82-1.04) | 0.95 (0.84-1.06) | 0.94 (0.83-1.06) |
| Q3 (50-53) | 454/381997 | 1.19 | 0.90 (0.79-1.02) | 0.93 (0.82-1.06) | 0.92 (0.81-1.05) |
| Q4 (54-77) | 549/500093 | 1.10 | **0.83 (0.74-0.94)** | **0.88 (0.78-0.99)** | **0.87 (0.77-0.99)** |
| *P* trend * |  |  | **0.0030** | **0.0408** | **0.0318** |
| Per 10 increases |  |  | **0.85 (0.78-0.92)** | **0.88 (0.81-0.95)** | **0.88 (0.81-0.96)** |
| ***hPDI*** |  |  |  |  |  |
| Q1 (29-50) | 548/438678 | 1.25 | 1.00 (ref.) | 1.00 (ref.) | 1.00 (ref.) |
| Q2 (51-54) | 568/451534 | 1.26 | 0.96 (0.85-1.08) | 0.97 (0.86-1.09) | 0.96 (0.85-1.08) |
| Q3 (55-58) | 546/458911 | 1.19 | 0.90 (0.79-1.01) | 0.91 (0.81-1.03) | 0.91 (0.81-1.03) |
| Q4 (59-82) | 501/465920 | 1.08 | **0.83 (0.73-0.94)** | **0.85 (0.75-0.97)** | **0.85 (0.75-0.97)** |
| *P* trend * |  |  | **0.0025** | **0.0105** | **0.0122** |
| Per 10 increases |  |  | **0.89 (0.83-0.96)** | **0.91 (0.84-0.98)** | **0.91 (0.84-0.99)** |
| ***uPDI*** |  |  |  |  |  |
| Q1 (28-51) | 478/405214 | 1.18 | 1.00 (ref.) | 1.00 (ref.) | 1.00 (ref.) |
| Q2 (52-55) | 605/453840 | 1.33 | **1.18 (1.05-1.33)** | **1.18 (1.05-1.34)** | **1.18 (1.04-1.33)** |
| Q3 (56-58) | 553/471695 | 1.17 | 1.10 (0.97-1.24) | 1.09 (0.97-1.24) | 1.08 (0.95-1.22) |
| Q4 (59-79) | 527/484295 | 1.09 | **1.17 (1.03-1.33)** | **1.15 (1.02-1.31)** | **1.14 (1.01-1.30)** |
| *P* trend * |  |  | 0.0622 | 0.1073 | 0.1542 |
| Per 10 increases |  |  | 1.06 (0.98-1.15) | 1.05 (0.97-1.13) | 1.05 (0.97-1.13) |

*Linear trend was tested by treating the plant-based diet index category as a continuous variable.

Model 1 adjusted for age (continuous), sex (female, male) and total energy intake (continuous).

Model 2 adjusted for Model 1 plus ethnicity (White, mixed, Asian, Black, Chinese, others, or unknown), education (college or university, vocational qualification, upper secondary, lower secondary, others, or unknown), Townsend deprivation index (in quintiles), body mass index (<18.5, 18.5-24.9, 25-29.9, or ≥30 kg/m^2^), alcohol frequency (daily or almost daily, 3 or 4 times a week, 1 or 2 times a week, 1 to 3 times a month, special occasions only, never, or unknown), smoking status (never, former, current, or unknown), and physical activity (low, moderate, high, or unknown).

Model 3 adjusted for Model 2 plus polygenic risk score for CRC (continuous), first 10 principal components of ancestry (in Units, continuous), and genotype measurement batch (continuous).

Abbreviations: CI, confidence interval; CRC, colorectal cancer; hPDI, healthful plant-based diet index; HR, hazard ratio; PDI, overall plant-based diet index; uPDI, unhealthful plant-based diet index.

**Table S8. Association between plant-based diet indices and risk of CRC incidence according to categories of genetic risk**

| **Plant-based diet indices** | **HR (95% CI)** | | | ***P* for interaction** |
| --- | --- | --- | --- | --- |
|  | **Low PRS**  **(N=58044)** | **Intermediate PRS**  **(N=58124)** | **High PRS**  **(N=58093)** |  |
| **PDI** |  |  |  | 0.9610 |
| Tertile 1 (24-47) | 1.00 (ref.) | 1.00 (ref.) | 1.00 (ref.) |  |
| Tertile 2 (48-52) | 0.82 (0.65-1.04) | 1.00 (0.83-1.20) | 0.99 (0.84-1.15) |  |
| Tertile 3 (53-77) | 0.86 (0.68-1.08) | **0.81 (0.67-0.99)** | 0.92 (0.79-1.08) |  |
| Per 10 increases | 0.85 (0.71-1.03) | 0.89 (0.77-1.03) | **0.88 (0.78-0.99)** |  |
| **hPDI** |  |  |  | 0.4912 |
| Tertile 1 (29-51) | 1.00 (ref.) | 1.00 (ref.) | 1.00 (ref.) |  |
| Tertile 2 (52-56) | 0.87 (0.69-1.09) | 0.91 (0.75-1.09) | 0.98 (0.84-1.14) |  |
| Tertile 3 (57-82) | **0.78 (0.61-0.999)** | 0.93 (0.76-1.13) | 0.87 (0.74-1.03) |  |
| Per 10 increases | 0.86 (0.72-1.03) | 0.94 (0.82-1.09) | 0.91 (0.81-1.02) |  |
| **uPDI** |  |  |  | 0.7381 |
| Tertile 1 (28-52) | 1.00 (ref.) | 1.00 (ref.) | 1.00 (ref.) |  |
| Tertile 2 (53-57) | 1.12 (0.89-1.40) | 0.97 (0.80-1.16) | 1.03 (0.88-1.19) |  |
| Tertile 3 (58-79) | 1.08 (0.85-1.38) | 1.10 (0.91-1.34) | 0.96 (0.81-1.12) |  |
| Per 10 increases | 1.05 (0.88-1.25) | 1.06 (0.92-1.22) | 1.03 (0.92-1.16) |  |

The models adjusted for age (continuous), sex (female, male), ethnicity (White, mixed, Asian, Black, Chinese, others, or unknown), education (college or university, vocational qualification, upper secondary, lower secondary, others, or unknown), Townsend deprivation index (in quintiles), body mass index (<18.5, 18.5-24.9, 25-29.9, or ≥30 kg/m2), alcohol frequency (daily or almost daily, 3 or 4 times a week, 1 or 2 times a week, 1 to 3 times a month, special occasions only, never, or unknown), smoking status (never, former, current, or unknown), physical activity (low, moderate, high, or unknown), total energy intake (continuous), first 10 principal components of ancestry (in Units, continuous), and genotype measurement batch (continuous).

Abbreviations: CI, confidence interval; CRC, colorectal cancer; hPDI, healthful plant-based diet index; HR, hazard ratio; PDI, overall plant-based diet index; PRS, polygenic risk score; uPDI, unhealthful plant-based diet index.

**Table S9. Subgroup analysis for the association between plant-based diet indices and risk of CRC incidence by sex**

| **Plant-based diet indices** | **Cases/person-years** | **Incident rate per 1000 person-years** | **HR (95% CI)** | | |
| --- | --- | --- | --- | --- | --- |
|  |  |  | **Model 1** | **Model 2** | **Model 3** |
| **Female (n=101253)** | | | | | |
| ***PDI*** |  |  |  |  |  |
| Q1 (24-46) | 183/201954 | 0.91 | 1.00 (ref.) | 1.00 (ref.) | 1.00 (ref.) |
| Q2 (47-49) | 238/268630 | 0.89 | 0.93 (0.76-1.12) | 0.94 (0.78-1.14) | 0.92 (0.75-1.12) |
| Q3 (50-53) | 211/218698 | 0.96 | 0.98 (0.80-1.19) | 1.00 (0.81-1.22) | 0.96 (0.79-1.18) |
| Q4 (54-77) | 296/300063 | 0.99 | 0.98 (0.81-1.19) | 1.01 (0.83-1.22) | 0.99 (0.81-1.20) |
| *P* trend * |  |  | 0.9258 | 0.7166 | 0.8647 |
| Per 10 increases |  |  | 0.98 (0.86-1.11) | 0.99 (0.87-1.13) | 0.99 (0.87-1.13) |
| ***hPDI*** |  |  |  |  |  |
| Q1 (29-50) | 165/187845 | 0.88 | 1.00 (ref.) | 1.00 (ref.) | 1.00 (ref.) |
| Q2 (51-54) | 229/234020 | 0.98 | 1.03 (0.84-1.26) | 1.04 (0.85-1.27) | 1.02 (0.83-1.26) |
| Q3 (55-58) | 253/264279 | 0.96 | 0.98 (0.80-1.20) | 0.98 (0.80-1.20) | 0.98 (0.80-1.21) |
| Q4 (59-82) | 281/303201 | 0.93 | 0.94 (0.77-1.15) | 0.94 (0.77-1.15) | 0.94 (0.77-1.16) |
| *P* trend * |  |  | 0.4166 | 0.3873 | 0.4524 |
| Per 10 increases |  |  | 0.94 (0.84-1.06) | 0.94 (0.83-1.06) | 0.94 (0.83-1.06) |
| ***uPDI*** |  |  |  |  |  |
| Q1 (28-51) | 201/228527 | 0.88 | 1.00 (ref.) | 1.00 (ref.) | 1.00 (ref.) |
| Q2 (52-55) | 282/251886 | 1.12 | **1.35 (1.12-1.62)** | **1.38 (1.15-1.65)** | **1.37 (1.14-1.65)** |
| Q3 (56-58) | 228/255980 | 0.89 | 1.14 (0.94-1.38) | 1.18 (0.97-1.43) | 1.16 (0.96-1.41) |
| Q4 (59-79) | 217/252952 | 0.86 | **1.27 (1.04-1.54)** | **1.31 (1.08-1.60)** | **1.29 (1.05-1.58)** |
| *P* trend * |  |  | 0.0570 | **0.0472** | 0.0795 |
| Per 10 increases |  |  | 1.11 (0.99-1.25) | 1.12 (0.99-1.26) | 1.11 (0.98-1.25) |
| **Male (n=85422)** | | | | | |
| ***PDI*** |  |  |  |  |  |
| Q1 (24-46) | 372/231827 | 1.60 | 1.00 (ref.) | 1.00 (ref.) | 1.00 (ref.) |
| Q2 (47-49) | 367/230540 | 1.59 | 0.93 (0.81-1.08) | 0.96 (0.83-1.11) | 0.96 (0.83-1.11) |
| Q3 (50-53) | 243/163298 | 1.49 | 0.86 (0.73-1.01) | 0.90 (0.76-1.06) | 0.91 (0.77-1.07) |
| Q4 (54-77) | 253/200030 | 1.26 | **0.72 (0.61-0.85)** | **0.78 (0.66-0.92)** | **0.78 (0.66-0.92)** |
| *P* trend * |  |  | **<0.0001** | **0.0024** | **0.0028** |
| Per 10 increases |  |  | **0.77 (0.69-0.85)** | **0.81 (0.73-0.90)** | **0.81 (0.73-0.90)** |
| ***hPDI*** |  |  |  |  |  |
| Q1 (29-50) | 383/250833 | 1.53 | 1.00 (ref.) | 1.00 (ref.) | 1.00 (ref.) |
| Q2 (51-54) | 339/217514 | 1.56 | 0.92 (0.80-1.07) | 0.94 (0.81-1.09) | 0.93 (0.80-1.08) |
| Q3 (55-58) | 293/194631 | 1.51 | 0.85 (0.73-1.01) | 0.88 (0.75-1.02) | 0.87 (0.74-1.02) |
| Q4 (59-82) | 220/162718 | 1.35 | **0.76 (0.64-0.90)** | **0.80 (0.67-0.95)** | **0.79 (0.67-0.95)** |
| *P* trend * |  |  | **0.0011** | **0.0070** | **0.0069** |
| Per 10 increases |  |  | **0.86 (0.77-0.95)** | **0.88 (0.80-0.98)** | **0.88 (0.80-0.98)** |
| ***uPDI*** |  |  |  |  |  |
| Q1 (28-51) | 277/176686 | 1.57 | 1.00 (ref.) | 1.00 (ref.) | 1.00 (ref.) |
| Q2 (52-55) | 323/201953 | 1.60 | 1.06 (0.90-1.24) | 1.05 (0.89-1.24) | 1.05 (0.89-1.23) |
| Q3 (56-58) | 325/215714 | 1.51 | 1.05 (0.90-1.24) | 1.03 (0.88-1.22) | 1.02 (0.87-1.20) |
| Q4 (59-79) | 310/231342 | 1.34 | 1.08 (0.91-1.27) | 1.05 (0.89-1.24) | 1.05 (0.89-1.24) |
| *P* trend * |  |  | 0.4094 | 0.6141 | 0.6586 |
| Per 10 increases |  |  | 1.03 (0.93-1.14) | 1.01 (0.91-1.12) | 1.01 (0.91-1.12) |

*Linear trend was tested by treating the plant-based diet index category as a continuous variable.

Model 1 adjusted for age (continuous) and total energy intake (continuous).

Model 2 adjusted for Model 1 plus ethnicity (White, mixed, Asian, Black, Chinese, others, or unknown), education (college or university, vocational qualification, upper secondary, lower secondary, others, or unknown), Townsend deprivation index (in quintiles), body mass index (<18.5, 18.5-24.9, 25-29.9, or ≥30 kg/m^2^), alcohol frequency (daily or almost daily, 3 or 4 times a week, 1 or 2 times a week, 1 to 3 times a month, special occasions only, never, or unknown), smoking status (never, former, current, or unknown), and physical activity (low, moderate, high, or unknown).

Model 3 adjusted for Model 2 plus polygenic risk score for CRC (continuous), first 10 principal components of ancestry (in Units, continuous), and genotype measurement batch (continuous).

Abbreviations: CI, confidence interval; CRC, colorectal cancer; hPDI, healthful plant-based diet index; HR, hazard ratio; PDI, overall plant-based diet index; uPDI, unhealthful plant-based diet index.

**Table S10. Subgroup analysis for the association between PDI and risk of CRC incidence**

| **Subgroups** | **N** | **HR (95% CI)** | | | | ***P* for interaction** |
| --- | --- | --- | --- | --- | --- | --- |
|  |  | **Q1** | **Q2** | **Q3** | **Q4** |  |
| **Age** |  |  |  |  |  |  |
| <60 years | 113930 | 1.00 (ref.) | 0.98 (0.80-1.20) | 0.98 (0.78-1.22) | 1.00 (0.81-1.23) | 0.3953 |
| ≥60 years | 87860 | 1.00 (ref.) | 0.96 (0.83-1.10) | 0.95 (0.81-1.10) | 0.87 (0.75-1.01) |  |
| **Townsend deprivation index** |  |  |  |  |  |  |
| Below median value | 93220 | 1.00 (ref.) | 0.96 (0.82-1.12) | 0.92 (0.78-1.10) | **0.80 (0.68-0.95)** | 0.0652 |
| Above median value | 93220 | 1.00 (ref.) | 0.94 (0.79-1.12) | 0.95 (0.79-1.15) | 0.98 (0.82-1.17) |  |
| **Body mass index** |  |  |  |  |  |  |
| <25 | 69614 | 1.00 (ref.) | 0.81 (0.65-1.01) | 0.82 (0.65-1.03) | **0.77 (0.62-0.96)** | 0.5657 |
| 25-29.9 | 77491 | 1.00 (ref.) | 1.01 (0.85-1.20) | 1.04 (0.87-1.26) | 0.91 (0.76-1.09) |  |
| ≥30 | 39058 | 1.00 (ref.) | 1.02 (0.81-1.27) | 0.89 (0.68-1.15) | 0.97 (0.75-1.24) |  |
| **Alcohol frequency** |  |  |  |  |  |  |
| ≥3 or 4 times a week | 89379 | 1.00 (ref.) | 0.93 (0.80-1.09) | 0.85 (0.72-1.01) | **0.84 (0.71-0.99)** | 0.2023 |
| <3 or 4 times a week | 97137 | 1.00 (ref.) | 0.98 (0.82-1.17) | 1.05 (0.87-1.27) | 0.95 (0.79-1.14) |  |
| **Smoking status** |  |  |  |  |  |  |
| Never | 106261 | 1.00 (ref.) | 0.97 (0.82-1.16) | 0.94 (0.78-1.13) | 0.92 (0.77-1.10) | 0.4338 |
| Former smokers | 65351 | 1.00 (ref.) | 0.92 (0.77-1.09) | 0.95 (0.79-1.14) | 0.87 (0.73-1.04) |  |
| Current smokers | 14577 | 1.00 (ref.) | 1.08 (0.75-1.56) | 0.98 (0.63-1.51) | 0.74 (0.47-1.18) |  |
| **Physical activity** |  |  |  |  |  |  |
| Low | 29121 | 1.00 (ref.) | 1.12 (0.85-1.47) | 1.24 (0.92-1.68) | 1.03 (0.76-1.40) | 0.4415 |
| Moderate | 66988 | 1.00 (ref.) | 0.88 (0.73-1.07) | 0.90 (0.74-1.11) | **0.81 (0.66-0.99)** |  |
| High | 62321 | 1.00 (ref.) | 1.02 (0.82-1.26) | 0.86 (0.68-1.09) | 0.88 (0.71-1.09) |  |

The models adjusted for age (continuous), sex (female, male), ethnicity (White, mixed, Asian, Black, Chinese, others, or unknown), education (college or university, vocational qualification, upper secondary, lower secondary, others, or unknown), Townsend deprivation index (in quintiles), body mass index (<18.5, 18.5-24.9, 25-29.9, or ≥30 kg/m^2^), alcohol frequency (daily or almost daily, 3 or 4 times a week, 1 or 2 times a week, 1 to 3 times a month, special occasions only, never, or unknown), smoking status (never, former, current, or unknown), physical activity (low, moderate, high, or unknown), total energy intake (continuous), polygenic risk score for CRC (continuous), first 10 principal components of ancestry (in Units, continuous), and genotype measurement batch (continuous).

Abbreviations: CI, confidence interval; CRC, colorectal cancer; HR, hazard ratio; PDI, overall plant-based diet index.

**Table S11. Subgroup analysis for the association between hPDI and risk of CRC incidence**

| **Subgroups** | **N** | **HR (95% CI)** | | | | ***P* for interaction** |
| --- | --- | --- | --- | --- | --- | --- |
|  |  | **Q1** | **Q2** | **Q3** | **Q4** |  |
| **Age** |  |  |  |  |  |  |
| <60 years | 113930 | 1.00 (ref.) | 1.03 (0.84-1.27) | 0.87 (0.70-1.09) | 1.20 (0.96-1.49) | **0.0238** |
| ≥60 years | 87860 | 1.00 (ref.) | 0.97 (0.84-1.12) | 0.96 (0.83-1.11) | **0.76 (0.65-0.90)** |  |
| **Townsend deprivation index** |  |  |  |  |  |  |
| Below median value | 93220 | 1.00 (ref.) | 0.98 (0.83-1.15) | 0.90 (0.76-1.07) | **0.83 (0.70-0.998)** | 0.7178 |
| Above median value | 93220 | 1.00 (ref.) | 0.95 (0.80-1.13) | 0.91 (0.76-1.09) | 0.85 (0.71-1.03) |  |
| **Body mass index** |  |  |  |  |  |  |
| <25 | 69614 | 1.00 (ref.) | 1.04 (0.82-1.31) | 0.92 (0.73-1.16) | 0.85 (0.67-1.07) | 0.9303 |
| 25-29.9 | 77491 | 1.00 (ref.) | 0.93 (0.78-1.10) | 0.94 (0.79-1.13) | **0.82 (0.67-0.99)** |  |
| ≥30 | 39058 | 1.00 (ref.) | 0.99 (0.78-1.24) | 0.81 (0.63-1.04) | 0.90 (0.69-1.17) |  |
| **Alcohol frequency** |  |  |  |  |  |  |
| ≥3 or 4 times a week | 89379 | 1.00 (ref.) | 0.96 (0.82-1.13) | 0.95 (0.81-1.12) | 0.88 (0.73-1.04) | 0.4999 |
| <3 or 4 times a week | 97137 | 1.00 (ref.) | 0.97 (0.81-1.15) | 0.85 (0.70-1.02) | **0.80 (0.66-0.97)** |  |
| **Smoking status** |  |  |  |  |  |  |
| Never | 106261 | 1.00 (ref.) | 0.97 (0.82-1.16) | 0.87 (0.73-1.04) | **0.76 (0.63-0.92)** | 0.4628 |
| Former smokers | 65351 | 1.00 (ref.) | 0.92 (0.77-1.11) | 0.94 (0.79-1.13) | 0.91 (0.76-1.10) |  |
| Current smokers | 14577 | 1.00 (ref.) | 1.24 (0.84-1.83) | 0.84 (0.54-1.31) | 0.99 (0.62-1.57) |  |
| **Physical activity** |  |  |  |  |  |  |
| Low | 29121 | 1.00 (ref.) | 0.90 (0.68-1.19) | 0.93 (0.69-1.24) | 0.90 (0.66-1.23) | 0.7604 |
| Moderate | 66988 | 1.00 (ref.) | 1.02 (0.84-1.24) | 0.92 (0.75-1.13) | 0.84 (0.68-1.04) |  |
| High | 62321 | 1.00 (ref.) | 1.03 (0.83-1.29) | 0.97 (0.77-1.21) | 0.83 (0.66-1.05) |  |

The models adjusted for age (continuous), sex (female, male), ethnicity (White, mixed, Asian, Black, Chinese, others, or unknown), education (college or university, vocational qualification, upper secondary, lower secondary, others, or unknown), Townsend deprivation index (in quintiles), body mass index (<18.5, 18.5-24.9, 25-29.9, or ≥30 kg/m^2^), alcohol frequency (daily or almost daily, 3 or 4 times a week, 1 or 2 times a week, 1 to 3 times a month, special occasions only, never, or unknown), smoking status (never, former, current, or unknown), physical activity (low, moderate, high, or unknown), total energy intake (continuous), polygenic risk score for CRC (continuous), first 10 principal components of ancestry (in Units, continuous), and genotype measurement batch (continuous).

Abbreviations: CI, confidence interval; CRC, colorectal cancer; hPDI, healthful plant-based diet index; HR, hazard ratio.

**Table S12. Subgroup analysis for the association between uPDI and risk of CRC incidence**

| **Subgroups** | **N** | **HR (95% CI)** | | | | ***P* for interaction** |
| --- | --- | --- | --- | --- | --- | --- |
|  |  | **Q1** | **Q2** | **Q3** | **Q4** |  |
| **Age** |  |  |  |  |  |  |
| <60 years | 113930 | 1.00 (ref.) | 1.12 (0.89-1.41) | 1.00 (0.79-1.25) | 0.95 (0.76-1.19) | 0.2052 |
| ≥60 years | 87860 | 1.00 (ref.) | **1.19 (1.04-1.37)** | 1.09 (0.94-1.26) | 1.14 (0.98-1.33) |  |
| **Townsend deprivation index** |  |  |  |  |  |  |
| Below median value | 93220 | 1.00 (ref.) | 1.16 (0.99-1.37) | 1.10 (0.93-1.31) | **1.26 (1.06-1.50)** | 0.0825 |
| Above median value | 93220 | 1.00 (ref.) | **1.21 (1.02-1.45)** | 1.08 (0.90-1.30) | 1.04 (0.86-1.26) |  |
| **Body mass index** |  |  |  |  |  |  |
| <25 | 69614 | 1.00 (ref.) | **1.34 (1.08-1.66)** | **1.31 (1.05-1.64)** | 1.18 (0.92-1.50) | 0.7064 |
| 25-29.9 | 77491 | 1.00 (ref.) | 1.07 (0.89-1.27) | 1.01 (0.84-1.22) | 1.15 (0.96-1.39) |  |
| ≥30 | 39058 | 1.00 (ref.) | 1.27 (0.99-1.63) | 1.00 (0.77-1.30) | 1.13 (0.88-1.47) |  |
| **Alcohol frequency** |  |  |  |  |  |  |
| ≥3 or 4 times a week | 89379 | 1.00 (ref.) | 1.11 (0.94-1.30) | 1.00 (0.85-1.18) | 1.05 (0.89-1.25) | 0.0887 |
| <3 or 4 times a week | 97137 | 1.00 (ref.) | **1.31 (1.09-1.57)** | **1.25 (1.04-1.50)** | **1.32 (1.09-1.59)** |  |
| **Smoking status** |  |  |  |  |  |  |
| Never | 106261 | 1.00 (ref.) | **1.25 (1.05-1.50)** | 1.20 (0.99-1.44) | **1.32 (1.10-1.60)** | 0.2209 |
| Former smokers | 65351 | 1.00 (ref.) | 1.12 (0.94-1.34) | 1.00 (0.84-1.20) | 0.99 (0.82-1.20) |  |
| Current smokers | 14577 | 1.00 (ref.) | 1.29 (0.81-2.05) | 1.22 (0.77-1.94) | 1.33 (0.84-2.10) |  |
| **Physical activity** |  |  |  |  |  |  |
| Low | 29121 | 1.00 (ref.) | 0.97 (0.71-1.31) | 0.93 (0.69-1.26) | 0.96 (0.71-1.29) | 0.5406 |
| Moderate | 66988 | 1.00 (ref.) | 1.16 (0.95-1.41) | 1.14 (0.93-1.39) | 1.12 (0.91-1.39) |  |
| High | 62321 | 1.00 (ref.) | 1.17 (0.95-1.44) | 1.05 (0.84-1.30) | 1.18 (0.95-1.48) |  |

The models adjusted for age (continuous), sex (female, male), ethnicity (White, mixed, Asian, Black, Chinese, others, or unknown), education (college or university, vocational qualification, upper secondary, lower secondary, others, or unknown), Townsend deprivation index (in quintiles), body mass index (<18.5, 18.5-24.9, 25-29.9, or ≥30 kg/m^2^), alcohol frequency (daily or almost daily, 3 or 4 times a week, 1 or 2 times a week, 1 to 3 times a month, special occasions only, never, or unknown), smoking status (never, former, current, or unknown), physical activity (low, moderate, high, or unknown), total energy intake (continuous), polygenic risk score for CRC (continuous), first 10 principal components of ancestry (in Units, continuous), and genotype measurement batch (continuous).

Abbreviations: CI, confidence interval; CRC, colorectal cancer; HR, hazard ratio; uPDI, unhealthful plant-based diet index.

**Table S13. Association between plant-based diet indices and risk of CRC mortality**

| **Plant-based diet indices** | **Deaths/person-years** | **Mortality rate per 1000 person-years** | **HR (95% CI)** | | |
| --- | --- | --- | --- | --- | --- |
|  |  |  | **Model 1** | **Model 2** | **Model 3** |
| ***PDI*** |  |  |  |  |  |
| Q1 (24-46) | 126/442897 | 0.28 | 1.00 (ref.) | 1.00 (ref.) | 1.00 (ref.) |
| Q2 (47-49) | 131/509828 | 0.26 | 0.90 (0.71-1.15) | 0.93 (0.72-1.19) | 0.91 (0.71-1.17) |
| Q3 (50-53) | 106/390194 | 0.27 | 0.96 (0.74-1.25) | 1.02 (0.78-1.32) | 1.00 (0.77-1.30) |
| Q4 (54-77) | 103/510773 | 0.20 | **0.74 (0.57-0.97)** | 0.79 (0.60-1.04) | 0.77 (0.59-1.01) |
| *P* trend * |  |  | **0.0496** | 0.1563 | 0.1172 |
| Per 10 increases |  |  | **0.81 (0.68-0.96)** | 0.85 (0.71-1.01) | 0.84 (0.70-1.01) |
| ***hPDI*** |  |  |  |  |  |
| Q1 (29-50) | 122/448155 | 0.27 | 1.00 (ref.) | 1.00 (ref.) | 1.00 (ref.) |
| Q2 (51-54) | 114/461461 | 0.25 | 0.83 (0.64-1.07) | 0.85 (0.66-1.10) | 0.84 (0.64-1.09) |
| Q3 (55-58) | 118/468689 | 0.25 | 0.82 (0.63-1.07) | 0.85 (0.66-1.11) | 0.86 (0.66-1.13) |
| Q4 (59-82) | 112/475387 | 0.24 | 0.77 (0.59-1.01) | 0.80 (0.61-1.05) | 0.80 (0.60-1.05) |
| *P* trend * |  |  | 0.0775 | 0.1331 | 0.1564 |
| Per 10 increases |  |  | 0.86 (0.73-1.02) | 0.88 (0.75-1.05) | 0.89 (0.75-1.05) |
| ***uPDI*** |  |  |  |  |  |
| Q1 (28-51) | 105/413878 | 0.25 | 1.00 (ref.) | 1.00 (ref.) | 1.00 (ref.) |
| Q2 (52-55) | 131/463557 | 0.28 | 1.14 (0.88-1.48) | 1.15 (0.89-1.49) | 1.16 (0.89-1.51) |
| Q3 (56-58) | 123/481826 | 0.26 | 1.07 (0.83-1.39) | 1.08 (0.83-1.40) | 1.10 (0.84-1.44) |
| Q4 (59-79) | 107/494431 | 0.22 | 1.03 (0.78-1.35) | 1.02 (0.77-1.34) | 1.01 (0.76-1.34) |
| *P* trend * |  |  | 0.9763 | 0.9334 | 0.9398 |
| Per 10 increases |  |  | 1.04 (0.88-1.22) | 1.03 (0.87-1.21) | 1.02 (0.86-1.21) |

*Linear trend was tested by treating the plant-based diet index category as a continuous variable.

Model 1 adjusted for age (continuous), sex (female, male) and total energy intake (continuous).

Model 2 adjusted for Model 1 plus ethnicity (White, mixed, Asian, Black, Chinese, others, or unknown), education (college or university, vocational qualification, upper secondary, lower secondary, others, or unknown), Townsend deprivation index (in quintiles), body mass index (<18.5, 18.5-24.9, 25-29.9, or ≥30 kg/m^2^), alcohol frequency (daily or almost daily, 3 or 4 times a week, 1 or 2 times a week, 1 to 3 times a month, special occasions only, never, or unknown), smoking status (never, former, current, or unknown), and physical activity (low, moderate, high, or unknown).

Model 3 adjusted for Model 2 plus polygenic risk score for CRC (continuous), first 10 principal components of ancestry (in Units, continuous), and genotype measurement batch (continuous).

Abbreviations: CI, confidence interval; CRC, colorectal cancer; hPDI, healthful plant-based diet index; HR, hazard ratio; PDI, overall plant-based diet index; uPDI, unhealthful plant-based diet index.

**Table S14. Subgroup analysis for the association between plant-based diet indices and risk of CRC mortality by sex**

| **Plant-based diet indices** | **Deaths/person-years** | **Mortality rate per 1000 person-years** | **HR (95% CI)** | | |
| --- | --- | --- | --- | --- | --- |
|  |  |  | **Model 1** | **Model 2** | **Model 3** |
| **Female (n=101253)** | | | | | |
| ***PDI*** |  |  |  |  |  |
| Q1 (24-46) | 40/206033 | 0.19 | 1.00 (ref.) | 1.00 (ref.) | 1.00 (ref.) |
| Q2 (47-49) | 48/273932 | 0.17 | 0.86 (0.57-1.32) | 0.90 (0.59-1.37) | 0.90 (0.59-1.37) |
| Q3 (50-53) | 41/223359 | 0.18 | 0.88 (0.57-1.37) | 0.92 (0.59-1.44) | 0.92 (0.59-1.44) |
| Q4 (54-77) | 58/306397 | 0.19 | 0.92 (0.61-1.39) | 0.97 (0.64-1.48) | 0.98 (0.64-1.49) |
| *P* trend * |  |  | 0.8075 | 0.9972 | 0.9847 |
| Per 10 increases |  |  | 0.94 (0.70-1.25) | 0.97 (0.72-1.29) | 0.97 (0.73-1.30) |
| ***hPDI*** |  |  |  |  |  |
| Q1 (29-50) | 27/191734 | 0.14 | 1.00 (ref.) | 1.00 (ref.) | 1.00 (ref.) |
| Q2 (51-54) | 48/238974 | 0.20 | 1.27 (0.79-2.05) | 1.28 (0.80-2.06) | 1.28 (0.80-2.06) |
| Q3 (55-58) | 52/269804 | 0.19 | 1.17 (0.73-1.88) | 1.16 (0.72-1.87) | 1.17 (0.73-1.88) |
| Q4 (59-82) | 60/309208 | 0.19 | 1.15 (0.72-1.84) | 1.12 (0.69-1.80) | 1.12 (0.70-1.80) |
| *P* trend * |  |  | 0.8143 | 0.9378 | 0.9289 |
| Per 10 increases |  |  | 1.04 (0.80-1.35) | 1.02 (0.78-1.33) | 1.02 (0.78-1.34) |
| ***uPDI*** |  |  |  |  |  |
| Q1 (28-51) | 43/233216 | 0.18 | 1.00 (ref.) | 1.00 (ref.) | 1.00 (ref.) |
| Q2 (52-55) | 64/257217 | 0.25 | 1.43 (0.97-2.10) | 1.47 (0.99-2.17) | 1.47 (0.99-2.17) |
| Q3 (56-58) | 51/261198 | 0.20 | 1.19 (0.79-1.79) | 1.23 (0.82-1.87) | 1.23 (0.82-1.86) |
| Q4 (59-79) | 29/258089 | 0.11 | 0.80 (0.50-1.29) | 0.82 (0.51-1.33) | 0.82 (0.51-1.33) |
| *P* trend * |  |  | 0.3251 | 0.3978 | 0.3887 |
| Per 10 increases |  |  | 0.89 (0.68-1.15) | 0.91 (0.70-1.19) | 0.91 (0.70-1.18) |
| **Male (n=85422)** | | | | | |
| ***PDI*** |  |  |  |  |  |
| Q1 (24-46) | 86/236964 | 0.36 | 1.00 (ref.) | 1.00 (ref.) | 1.00 (ref.) |
| Q2 (47-49) | 83/235896 | 0.35 | 0.93 (0.69-1.26) | 0.96 (0.70-1.30) | 0.96 (0.70-1.30) |
| Q3 (50-53) | 65/166835 | 0.39 | 1.03 (0.74-1.42) | 1.09 (0.79-1.51) | 1.09 (0.79-1.51) |
| Q4 (54-77) | 45/204376 | 0.22 | **0.59 (0.41-0.86)** | **0.64 (0.44-0.93)** | **0.64 (0.44-0.93)** |
| *P* trend * |  |  | **0.0183** | 0.0618 | 0.0620 |
| Per 10 increases |  |  | **0.74 (0.59-0.92)** | **0.78 (0.62-0.97)** | **0.78 (0.62-0.97)** |
| ***hPDI*** |  |  |  |  |  |
| Q1 (29-50) | 95/256420 | 0.37 | 1.00 (ref.) | 1.00 (ref.) | 1.00 (ref.) |
| Q2 (51-54) | 66/222486 | 0.30 | **0.68 (0.50-0.94)** | **0.71 (0.51-0.97)** | **0.71 (0.51-0.97)** |
| Q3 (55-58) | 66/198885 | 0.33 | **0.71 (0.51-0.98)** | 0.75 (0.54-1.03) | 0.75 (0.54-1.03) |
| Q4 (59-82) | 52/166179 | 0.31 | **0.64 (0.45-0.91)** | **0.68 (0.47-0.97)** | **0.68 (0.47-0.97)** |
| *P* trend * |  |  | **0.0139** | **0.0388** | **0.0388** |
| Per 10 increases |  |  | **0.76 (0.61-0.94)** | **0.80 (0.64-0.99)** | **0.80 (0.64-0.99)** |
| ***uPDI*** |  |  |  |  |  |
| Q1 (28-51) | 62/180662 | 0.34 | 1.00 (ref.) | 1.00 (ref.) | 1.00 (ref.) |
| Q2 (52-55) | 67/206340 | 0.32 | 0.96 (0.68-1.35) | 0.96 (0.68-1.36) | 0.96 (0.68-1.36) |
| Q3 (56-58) | 72/220628 | 0.33 | 1.00 (0.71-1.41) | 0.99 (0.70-1.40) | 0.99 (0.70-1.39) |
| Q4 (59-79) | 78/236341 | 0.33 | 1.15 (0.82-1.61) | 1.11 (0.79-1.56) | 1.11 (0.79-1.56) |
| *P* trend * |  |  | 0.3908 | 0.5178 | 0.5177 |
| Per 10 increases |  |  | 1.15 (0.93-1.42) | 1.12 (0.90-1.39) | 1.12 (0.90-1.39) |

*Linear trend was tested by treating the plant-based diet index category as a continuous variable.

Model 1 adjusted for age (continuous) and total energy intake (continuous).

Model 2 adjusted for Model 1 plus ethnicity (White, mixed, Asian, Black, Chinese, others, or unknown), education (college or university, vocational qualification, upper secondary, lower secondary, others, or unknown), Townsend deprivation index (in quintiles), body mass index (<18.5, 18.5-24.9, 25-29.9, or ≥30 kg/m^2^), alcohol frequency (daily or almost daily, 3 or 4 times a week, 1 or 2 times a week, 1 to 3 times a month, special occasions only, never, or unknown), smoking status (never, former, current, or unknown), and physical activity (low, moderate, high, or unknown).

Model 3 adjusted for Model 2 plus polygenic risk score for CRC (continuous), first 10 principal components of ancestry (in Units, continuous), and genotype measurement batch (continuous).

Abbreviations: CI, confidence interval; CRC, colorectal cancer; hPDI, healthful plant-based diet index; HR, hazard ratio; PDI, overall plant-based diet index; uPDI, unhealthful plant-based diet index.

**Table S15. Association between plant-based diet indices and risk of CRC mortality according to categories of genetic risk**

| **Plant-based diet indices** | **HR (95% CI)** | | | ***P* for interaction** |
| --- | --- | --- | --- | --- |
|  | **Low PRS**  **(N=58044)** | **Intermediate PRS**  **(N=58124)** | **High PRS**  **(N=58093)** |  |
| **PDI** |  |  |  | 0.7627 |
| Tertile 1 (24-47) | 1.00 (ref.) | 1.00 (ref.) | 1.00 (ref.) |  |
| Tertile 2 (48-52) | 0.71 (0.42-1.21) | 1.17 (0.78-1.75) | 0.91 (0.66-1.26) |  |
| Tertile 3 (53-77) | 0.67 (0.39-1.14) | 0.88 (0.57-1.35) | 0.85 (0.61-1.19) |  |
| Per 10 increases | 0.77 (0.51-1.17) | 0.97 (0.70-1.34) | 0.78 (0.60-1.01) |  |
| **hPDI** |  |  |  | 0.8152 |
| Tertile 1 (29-51) | 1.00 (ref.) | 1.00 (ref.) | 1.00 (ref.) |  |
| Tertile 2 (52-56) | 0.83 (0.49-1.41) | 0.93 (0.61-1.40) | 1.06 (0.78-1.46) |  |
| Tertile 3 (57-82) | 0.84 (0.48-1.48) | 0.92 (0.59-1.42) | 0.77 (0.54-1.11) |  |
| Per 10 increases | 0.84 (0.56-1.25) | 0.93 (0.68-1.27) | 0.84 (0.65-1.08) |  |
| **uPDI** |  |  |  | 0.1672 |
| Tertile 1 (28-52) | 1.00 (ref.) | 1.00 (ref.) | 1.00 (ref.) |  |
| Tertile 2 (53-57) | 1.05 (0.62-1.79) | 1.05 (0.69-1.58) | 1.06 (0.77-1.44) |  |
| Tertile 3 (58-79) | 1.26 (0.74-2.15) | 1.27 (0.84-1.94) | 0.78 (0.55-1.11) |  |
| Per 10 increases | 1.12 (0.76-1.67) | 1.16 (0.85-1.57) | 0.93 (0.73-1.19) |  |

The models adjusted for age (continuous), sex (female, male), ethnicity (White, mixed, Asian, Black, Chinese, others, or unknown), education (college or university, vocational qualification, upper secondary, lower secondary, others, or unknown), Townsend deprivation index (in quintiles), body mass index (<18.5, 18.5-24.9, 25-29.9, or ≥30 kg/m2), alcohol frequency (daily or almost daily, 3 or 4 times a week, 1 or 2 times a week, 1 to 3 times a month, special occasions only, never, or unknown), smoking status (never, former, current, or unknown), physical activity (low, moderate, high, or unknown), total energy intake (continuous), first 10 principal components of ancestry (in Units, continuous), and genotype measurement batch (continuous).

Abbreviations: CI, confidence interval; CRC, colorectal cancer; hPDI, healthful plant-based diet index; HR, hazard ratio; PDI, overall plant-based diet index; PRS, polygenic risk score; uPDI, unhealthful plant-based diet index.

**Table S16. Sensitivity analyses for the association between plant-based diet indices and risks of CRC incidence and mortality**

|  | **N** | **HR (95% CI)** | | | | ***P* trend*** |
| --- | --- | --- | --- | --- | --- | --- |
|  |  | **Q1** | **Q2** | **Q3** | **Q4** |  |
| ***Sensitivity analysis 1: excluding individuals with less than 2 years of follow-up*** | | | | | | |
| **For CRC incidence** |  |  |  |  |  |  |
| PDI | 185404 | 1.00 (ref.) | 0.95 (0.84-1.08) | 0.91 (0.79-1.04) | **0.86 (0.76-0.99)** | **0.0245** |
| hPDI | 185404 | 1.00 (ref.) | 1.05 (0.92-1.19) | 0.90 (0.78-1.03) | 0.89 (0.77-1.03) | **0.0241** |
| uPDI | 185404 | 1.00 (ref.) | **1.23 (1.08-1.40)** | **1.16 (1.02-1.33)** | 1.15 (0.99-1.32) | 0.1330 |
| **For CRC mortality** |  |  |  |  |  |  |
| PDI | 185711 | 1.00 (ref.) | 0.95 (0.74-1.22) | 1.01 (0.77-1.33) | 0.78 (0.59-1.03) | 0.1256 |
| hPDI | 185711 | 1.00 (ref.) | 0.87 (0.67-1.14) | 0.86 (0.66-1.13) | 0.80 (0.60-1.07) | 0.1428 |
| uPDI | 185711 | 1.00 (ref.) | 1.16 (0.88-1.51) | 1.09 (0.83-1.43) | 0.98 (0.73-1.30) | 0.7575 |
| ***Sensitivity analysis 2:*** ***using sub-distribution hazard models for competing risk*** | | | | | | |
| **For CRC incidence** |  |  |  |  |  |  |
| PDI | 186675 | 1.00 (ref.) | 0.95 (0.84-1.06) | 0.93 (0.82-1.06) | **0.88 (0.78-0.995)** | **0.0456** |
| hPDI | 186675 | 1.00 (ref.) | 0.97 (0.86-1.09) | 0.92 (0.81-1.03) | **0.86 (0.76-0.98)** | **0.0129** |
| uPDI | 186675 | 1.00 (ref.) | **1.18 (1.05-1.33)** | 1.09 (0.96-1.23) | **1.15 (1.01-1.30)** | 0.1216 |
| **For CRC mortality** |  |  |  |  |  |  |
| PDI | 186675 | 1.00 (ref.) | 0.93 (0.72-1.19) | 1.02 (0.78-1.33) | 0.79 (0.61-1.03) | 0.1508 |
| hPDI | 186675 | 1.00 (ref.) | 0.85 (0.66-1.10) | 0.86 (0.66-1.11) | 0.81 (0.61-1.06) | 0.1507 |
| uPDI | 186675 | 1.00 (ref.) | 1.15 (0.89-1.49) | 1.07 (0.82-1.40) | 1.01 (0.76-1.33) | 0.8869 |

*Linear trend was tested by treating the plant-based diet index category as a continuous variable.

The models adjusted for age (continuous), sex (female, male), ethnicity (White, mixed, Asian, Black, Chinese, others, or unknown), education (college or university, vocational qualification, upper secondary, lower secondary, others, or unknown), Townsend deprivation index (in quintiles), body mass index (<18.5, 18.5-24.9, 25-29.9, or ≥30 kg/m^2^), alcohol frequency (daily or almost daily, 3 or 4 times a week, 1 or 2 times a week, 1 to 3 times a month, special occasions only, never, or unknown), smoking status (never, former, current, or unknown), physical activity (low, moderate, high, or unknown), total energy intake (continuous), polygenic risk score for CRC (continuous), first 10 principal components of ancestry (in Units, continuous), and genotype measurement batch (continuous).

Abbreviations: CI, confidence interval; CRC, colorectal cancer; hPDI, healthful plant-based diet index; HR, hazard ratio; PDI, overall plant-based diet index; uPDI, unhealthful plant-based diet index.

**Table S17. Association between 3 food categories and risks of CRC incidence and mortality**

|  | **HR (95% CI)** | | | | | ***P* trend*** |
| --- | --- | --- | --- | --- | --- | --- |
|  | **Continuous** | **Q1** | **Q2** | **Q3** | **Q4** |  |
| **CRC incidence** | | | | | | |
| **All participants** |  |  |  |  |  |  |
| Healthy plant food groups | **0.94 (0.90-0.98)** | 1.00 (ref.) | 0.91 (0.81-1.02) | 0.91 (0.80-1.04) | **0.82 (0.72-0.92)** | **0.0024** |
| Less healthy plant food groups | 0.99 (0.95-1.04) | 1.00 (ref.) | 1.05 (0.92-1.18) | 1.05 (0.93-1.18) | 0.97 (0.84-1.12) | 0.8049 |
| Animal food groups | 1.02 (0.98-1.07) | 1.00 (ref.) | 1.05 (0.92-1.20) | 1.00 (0.88-1.14) | 1.08 (0.95-1.24) | 0.3504 |
| **Female** |  |  |  |  |  |  |
| Healthy plant food groups | 0.97 (0.91-1.03) | 1.00 (ref.) | 0.92 (0.76-1.12) | 0.94 (0.76-1.15) | 0.89 (0.73-1.08) | 0.3016 |
| Less healthy plant food groups | 1.03 (0.96-1.10) | 1.00 (ref.) | 1.19 (0.99-1.43) | 1.10 (0.91-1.32) | 1.14 (0.91-1.42) | 0.3999 |
| Animal food groups | 0.97 (0.91-1.04) | 1.00 (ref.) | 1.01 (0.84-1.21) | 0.87 (0.72-1.05) | 0.96 (0.79-1.18) | 0.3957 |
| **Male** |  |  |  |  |  |  |
| Healthy plant food groups | **0.92 (0.87-0.97)** | 1.00 (ref.) | 0.90 (0.78-1.05) | 0.90 (0.76-1.07) | **0.75 (0.64-0.89)** | **0.0014** |
| Less healthy plant food groups | 0.97 (0.92-1.03) | 1.00 (ref.) | 0.94 (0.79-1.11) | 1.01 (0.87-1.19) | 0.87 (0.72-1.04) | 0.3199 |
| Animal food groups | 1.06 (0.99-1.12) | 1.00 (ref.) | 1.08 (0.90-1.31) | 1.12 (0.94-1.35) | 1.19 (0.99-1.43) | 0.0555 |
| **CRC mortality** | | | | | | |
| **All participants** |  |  |  |  |  |  |
| Healthy plant food groups | 0.95 (0.87-1.03) | 1.00 (ref.) | 0.92 (0.71-1.18) | 1.01 (0.77-1.33) | 0.81 (0.62-1.07) | 0.2169 |
| Less healthy plant food groups | 0.98 (0.89-1.08) | 1.00 (ref.) | 0.98 (0.75-1.27) | 0.96 (0.75-1.24) | 0.94 (0.69-1.28) | 0.6883 |
| Animal food groups | 1.07 (0.98-1.17) | 1.00 (ref.) | 1.09 (0.82-1.45) | 1.13 (0.85-1.50) | 1.23 (0.92-1.64) | 0.1587 |
| **Female** |  |  |  |  |  |  |
| Healthy plant food groups | 1.03 (0.90-1.18) | 1.00 (ref.) | 1.08 (0.69-1.69) | 1.36 (0.86-2.16) | 1.07 (0.68-1.68) | 0.6905 |
| Less healthy plant food groups | 0.94 (0.81-1.10) | 1.00 (ref.) | 1.05 (0.71-1.56) | 0.94 (0.63-1.41) | 0.82 (0.49-1.38) | 0.4436 |
| Animal food groups | 1.02 (0.88-1.18) | 1.00 (ref.) | 1.11 (0.74-1.67) | 0.89 (0.58-1.38) | 1.15 (0.73-1.81) | 0.8089 |
| **Male** |  |  |  |  |  |  |
| Healthy plant food groups | **0.89 (0.80-0.99)** | 1.00 (ref.) | 0.87 (0.64-1.17) | 0.86 (0.61-1.23) | **0.69 (0.48-0.98)** | **0.0480** |
| Less healthy plant food groups | 1.01 (0.89-1.14) | 1.00 (ref.) | 0.93 (0.65-1.31) | 0.98 (0.70-1.37) | 1.02 (0.69-1.50) | 0.8879 |
| Animal food groups | 1.10 (0.98-1.24) | 1.00 (ref.) | 1.05 (0.70-1.56) | 1.31 (0.90-1.91) | 1.30 (0.89-1.90) | 0.1059 |

*Linear trend was tested by treating the plant-based diet index category as a continuous variable.

The models adjusted for age (continuous), sex (female, male), ethnicity (White, mixed, Asian, Black, Chinese, others, or unknown), education (college or university, vocational qualification, upper secondary, lower secondary, others, or unknown), Townsend deprivation index (in quintiles), body mass index (<18.5, 18.5-24.9, 25-29.9, or ≥30 kg/m^2^), alcohol frequency (daily or almost daily, 3 or 4 times a week, 1 or 2 times a week, 1 to 3 times a month, special occasions only, never, or unknown), smoking status (never, former, current, or unknown), physical activity (low, moderate, high, or unknown), total energy intake (continuous), polygenic risk score for CRC (continuous), first 10 principal components of ancestry (in Units, continuous), and genotype measurement batch (continuous).

Abbreviations: CI, confidence interval; CRC, colorectal cancer; hPDI, healthful plant-based diet index; HR, hazard ratio; PDI, overall plant-based diet index; uPDI, unhealthful plant-based diet index.

**Table S18. Association between the modified PDI/hPDI and risks of CRC incidence and mortality**

|  | **CRC incidence** | | **CRC mortality** | |
| --- | --- | --- | --- | --- |
|  | **Incident rate per 1000 person-years** | **HR (95% CI)** | **Mortality rate per 1000 person-years** | **HR (95% CI)** |
| ***The first analysis: Dairy products were given positive scores in modified PDI and hPDI.*** | | | | |
| **First modified PDI** |  |  |  |  |
| Q1 (25-46) | 1.26 | 1.00 (ref.) | 0.28 | 1.00 (ref.) |
| Q2 (47-50) | 1.25 | 0.97 (0.87-1.09) | 0.28 | 1.00 (0.79-1.27) |
| Q3 (51-54) | 1.20 | 0.92 (0.82-1.04) | 0.23 | 0.88 (0.68-1.14) |
| Q4 (55-75) | 1.03 | **0.80 (0.70-0.91)** | 0.21 | 0.81 (0.60-1.08) |
| *P* trend * |  | **0.0009** |  | 0.1007 |
| Per 10 increases |  | **0.84 (0.78-0.91)** |  | **0.83 (0.70-0.99)** |
| **First modified hPDI** |  |  |  |  |
| Q1 (28-50) | 1.23 | 1.00 (ref.) | 0.26 | 1.00 (ref.) |
| Q2 (51-54) | 1.32 | 1.02 (0.91-1.14) | 0.27 | 0.97 (0.75-1.24) |
| Q3 (55-58) | 1.16 | 0.89 (0.79-1.01) | 0.25 | 0.90 (0.70-1.17) |
| Q4 (59-81) | 1.06 | **0.83 (0.73-0.94)** | 0.23 | 0.85 (0.65-1.11) |
| *P* trend * |  | **0.0008** |  | 0.1979 |
| Per 10 increases |  | **0.88 (0.81-0.94)** |  | 0.87 (0.74-1.02) |
| ***The second analysis: Dairy and seafoods were given positive scores in modified PDI and hPDI.*** | | | | |
| **Second modified PDI** |  |  |  |  |
| Q1 (22-43) | 1.28 | 1.00 (ref.) | 0.28 | 1.00 (ref.) |
| Q2 (44-47) | 1.20 | 0.91 (0.81-1.03) | 0.26 | 0.93 (0.73-1.20) |
| Q3 (48-52) | 1.23 | 0.92 (0.82-1.04) | 0.26 | 0.95 (0.73-1.23) |
| Q4 (53-73) | 1.05 | **0.79 (0.69-0.89)** | 0.20 | 0.76 (0.57-1.01) |
| *P* trend * |  | **0.0007** |  | 0.0942 |
| Per 10 increases |  | **0.83 (0.77-0.90)** |  | **0.83 (0.71-0.98)** |
| **Second modified hPDI** |  |  |  |  |
| Q1 (24-47) | 1.22 | 1.00 (ref.) | 0.26 | 1.00 (ref.) |
| Q2 (48-52) | 1.35 | 1.03 (0.92-1.16) | 0.28 | 1.02 (0.79-1.31) |
| Q3 (53-56) | 1.15 | **0.88 (0.79-0.99)** | 0.23 | 0.84 (0.65-1.08) |
| Q4 (57-79) | 1.05 | **0.82 (0.72-0.93)** | 0.24 | 0.90 (0.68-1.18) |
| *P* trend * |  | **0.0003** |  | 0.2183 |
| Per 10 increases |  | **0.87 (0.81-0.93)** |  | 0.88 (0.75-1.02) |

*Linear trend was tested by treating the plant-based diet index category as a continuous variable.

The models adjusted for age (continuous), sex (female, male), ethnicity (White, mixed, Asian, Black, Chinese, others, or unknown), education (college or university, vocational qualification, upper secondary, lower secondary, others, or unknown), Townsend deprivation index (in quintiles), body mass index (<18.5, 18.5-24.9, 25-29.9, or ≥30 kg/m^2^), alcohol frequency (daily or almost daily, 3 or 4 times a week, 1 or 2 times a week, 1 to 3 times a month, special occasions only, never, or unknown), smoking status (never, former, current, or unknown), physical activity (low, moderate, high, or unknown), total energy intake (continuous), polygenic risk score for CRC (continuous), first 10 principal components of ancestry (in Units, continuous), and genotype measurement batch (continuous).

Abbreviations: CI, confidence interval; CRC, colorectal cancer; hPDI, healthful plant-based diet index; HR, hazard ratio; PDI, overall plant-based diet index.
